# Azobenzene-Modified Temperature-Responsive Short Elastin-like Peptides for Photo-Controlled Phase Transition

**DOI:** 10.1021/acssynbio.4c00889

**Published:** 2025-07-24

**Authors:** Keitaro Suyama, Elissa Ngoc Mai, Iori Maeda, Takeru Nose

**Affiliations:** † Faculty of Arts and Science, 12923 Kyushu University, Fukuoka 819-0395, Japan; ‡ Department of Chemistry, Faculty and Graduate School of Science, Kyushu University, Fukuoka 819-0395, Japan; § Department of Physics and Information Technology, 154441 Kyushu Institute of Technology, Iizuka, Fukuoka 820-8502, Japan

**Keywords:** elastin-like peptide, photoresponsive peptide, phase separation, temperature-responsive peptide, repetitive sequence

## Abstract

Elastin-like peptides (ELPs) have attracted attention as temperature-responsive biomaterials that can be used as drug carriers. In this study, temperature- and photoresponsive self-assembling peptide analogues were developed by conjugating short ELPs (total 20 amino acid residues) and azobenzene derivatives. The synthesized ELP–azobenzene conjugates exhibited reversible spectral changes upon UV or visible-light irradiation and temperature-responsive phase separation in aqueous solutions. The aggregation ability of the *trans*-isomers of the ELP–azobenzene conjugates was stronger than that of the *cis*-isomers. This phenomenon was attributed to the change in the hydrophilicity of the azobenzene moiety associated with photoisomerization from the *trans*- to the *cis*-isomer. In addition, aggregates of the ELP–azobenzene conjugate could be controlled by light irradiation. Therefore, this study provides a methodology for photo- and temperature-responsive ELP analogues with low molecular weights that can be easily synthesized by simple chemical reactions and are potential candidates for drug carriers that enable precise control of drug release.

## Graphical abstract

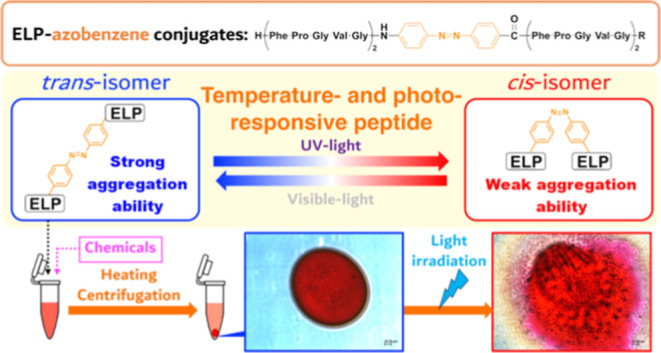

## 1 Introduction

In recent years, the development of stimulus-responsive nanocarriers made from various materials has advanced, enabling progress in delivery systems.^1,2^ To improve the efficacy and reduce the side effects of chemotherapy, drug nanocarriers that can specifically target the affected areas in the body or inhibit the rapid metabolism or excretion of drugs are required. In this context, the development of stimulus-responsive nanoparticles that can change their structures and functions in response to physicochemical factors (such as temperature,^3–5^ pH,^6–8^ light,^9–11^ redox,^12–14^ and enzymes^15^) has been actively pursued, especially for biomedical applications.^16–20^ Among these, temperature-responsive nanoparticles have been commonly utilized as drug carriers because the local body temperature can change depending on ambient conditions or diseases in several cases. Typical examples of the thermoresponsive nanoparticles include poly­(*N*-isopropylacrylamide) (PNIPAAm) derivatives,^21,22^ poly­(ethylene oxide)–poly­(propylene oxide) (PEO–PPO) pluronic copolymers,^23^ polymeric nanotubes,^24^ and micelles based on poly acrylamide derivatives.^25,26^ However, several synthetic polymers are often difficult to degrade in vivo, which limits their clinical applications.^27^ From this perspective, the development of biodegradable thermoresponsive materials composed of natural amino acids, particularly elastin-like peptides (ELPs), has attracted attention as a promising research strategy.^28–31^ ELPs are peptides artificially synthesized by mimicking the repetitive sequences of elastin, which is an extracellular matrix protein found in connective tissues.^32^ ELP analogues have been regarded as a class of peptide-based temperature-responsive materials that exhibit reversible phase separation in an aqueous environment in response to temperature change. The representative ELP is composed of the repetitive sequence Val–Pro–Gly–Val–Gly (VPGVG), which is derived from the sequence found in the hydrophobic domain of elastin of vertebrate species.^33–35^ In addition, we previously reported that short ELPs, (FPGVG)_*n*_, composed of Phe–Pro–Gly–Val–Gly sequences, where the former Val residue of (VPGVG) was replaced with phenylalanine, exhibited strong aggregation ability even at low repeat numbers (*n* = 5).^36,37^ To date, several studies have revealed that the phase separation of ELPs can be controlled by various factors, including the amino acid sequence,^38–40^ number of hydrophobic amino acid residues,^39^ peptide chain length,^40–42^ peptide concentration,^41,42^ solution pH,^43–45^ and ionic strength.^46,47^ Furthermore, as ELPs are derived from natural elastin, they are biocompatible, biodegradable, and have low immunogenicity.^48–50^ Thus, ELPs have been employed in various biomedical applications, including drug delivery, disease treatment research studies, and tissue repair.^51–55^

Generally, ELPs are used as drug carriers by utilizing the lower critical solution temperature (LCST) behavior of polymers, where the solubility of polymer particles increases with decreasing temperature below the LCST. However, controlling the phase separation of ELP analogues by lowering the temperature cannot be easily achieved in vivo or in medical applications. Therefore, as another possible external stimulus, investigation into controlling the phase separation of ELP with light stimulation was initiated. Light stimulation is widely used as a common external stimulus because it is readily available and offers a highly efficient stimulus with remote and precise spatiotemporal control. Recently, several studies have reported the development of photoresponsive ELP-based nanoparticles. For example, Le et al. reported diblock ELP-based nanoparticles that can rapidly switch their size upon irradiation with near-infrared-light.^56^ Furthermore, Amiram et al. developed photoresponsive ELPs containing azobenzene- or arylazopyrazole-bearing unnatural amino acids, the phase transition temperatures of which could be significantly and rapidly changed by light irradiation, by utilizing a high-performance orthogonal aminoacyl-tRNA synthetase.^57,58^ In this study, we describe the development of photoswitchable short ELP analogues by combining (FPGVG)_*n*_ peptides and azobenzene, which is the most robust and widely used class of photoswitch. Azobenzene derivatives undergo isomerization from the *trans*- to *cis*-isomer in response to UV-light irradiation and have been used for the photocontrol of biomolecules because of their highly efficient photochemical reactivity, biocompatibility of transition wavelengths, and low toxicity both before and after irradiation.^59,60^ In addition to the drastic structural change, the hydrophilicity of azobenzene is improved in response to isomerization from the *trans* to the *cis* conformation.^57,58,61^ The aggregation ability of short-chain (FPGVG)_*n*_ peptides significantly changes depending on the alteration in the hydrophobicity of its substructure. Therefore, by combining short (FPGVG)_*n*_ sequences and azobenzene, both photo- and temperature-responsive ELP analogues with significantly lower molecular weights that can be easily synthesized via simple chemical reactions can be developed. Such ELP analogues, the phase transition temperatures of which can be manipulated by light irradiation, are potential candidates for drug carriers, enabling precise control of drug release.

Within this framework, we developed conjugates of azobenzene derivatives with short ELP, (FPGVG)_2_ (F2), and its analogues. Subsequently, the phase separation properties of the synthesized analogues were investigated using spectrophotometry, dynamic light scattering (DLS), and microscopy before and after irradiation with UV/visible light. Furthermore, changes in the structural properties of the ELP–azobenzene conjugates in response to light irradiation were examined by using molecular dynamics (MD) simulations.

## 2 Results and Discussion

### 2.1 Synthesis and Purification of Peptides

ELP–azobenzene conjugates were synthesized by condensing short ELP analogues with azobenzene analogues containing amino or carboxyl or both substituents on their aromatic rings. As short ELP analogues, H-(FPGVG)_2_-NH_2_ and its analogues were synthesized using a conventional solid-phase peptide synthesis procedure. The chemical structures of the synthesized ELP–azobenzene conjugates are shown in Figure 1. In this study, two types of conjugates were synthesized, which were referred to as linear and dimeric conjugates. Initially, two linear conjugates, F2-Azb-F2 and F2-Azb-F2-NH_2_, were synthesized. These peptides were designed to embed azobenzene into a linear peptide chain by using an azobenzene derivative with an amino group on one aromatic ring and a carboxyl group on the other (Figure 1A). C-terminal free and amide variants were synthesized to investigate the effect of changing the hydrophilicity of the peptide on the aggregation ability. However, these analogues showed low water solubility, probably because of the hydrophobic nature of azobenzene. To enhance the water solubility, several dimeric ELP–azobenzene conjugates with multiple carboxyl groups were synthesized by condensing the N or C termini of the two peptides with substituents on the aromatic rings of azobenzene (Figure 1B). Azb-2­(K-F2) and 2­(F2-D)-Azb were designed to contain multiple amino and carboxyl groups in their peptide chains to form zwitterions in phosphate-buffered saline (PBS).

**Figure 1:**
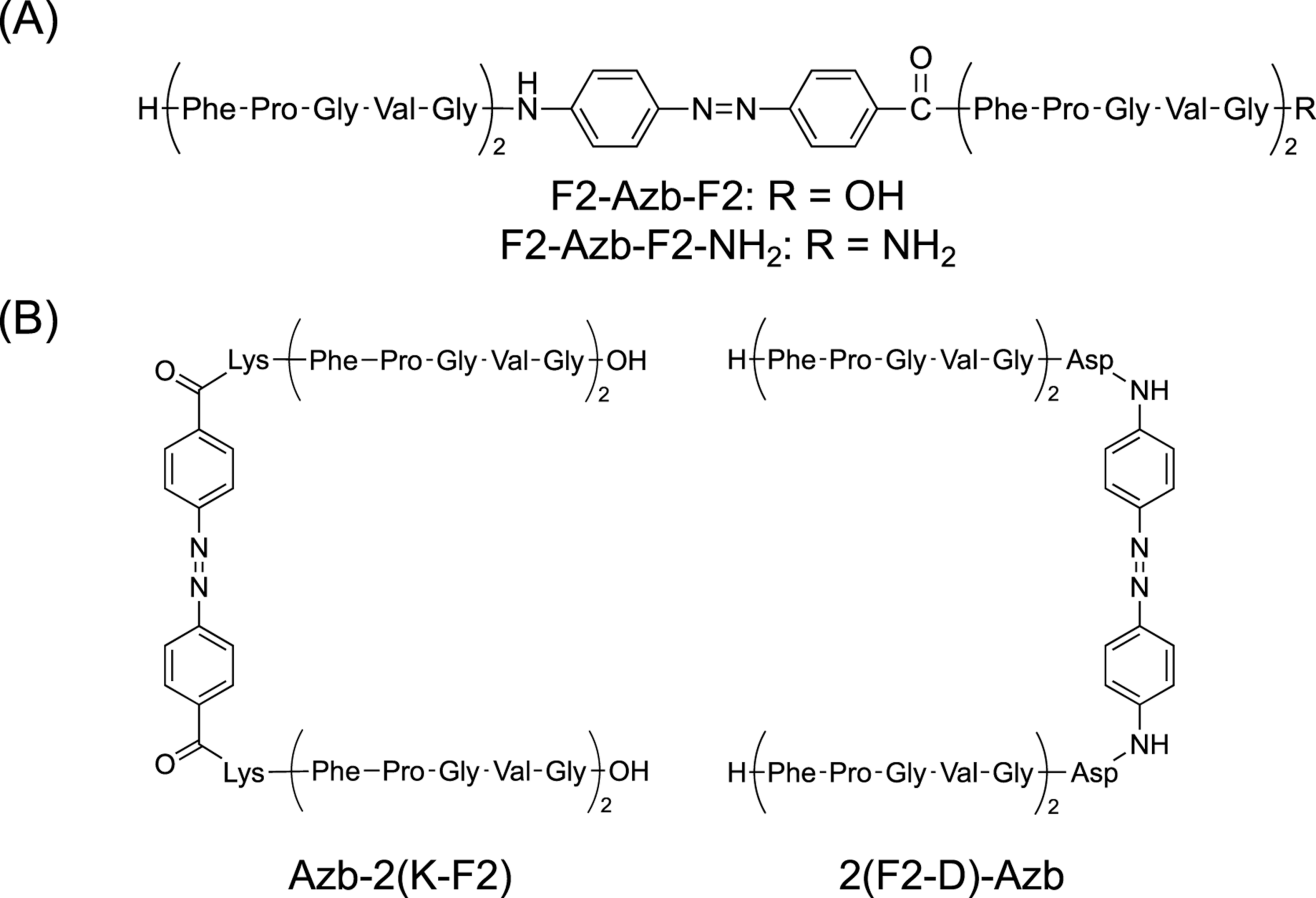
Structures of the short ELP–azobenzene conjugate analogues developed in this study. (A) The structure of the linear ELP–azobenzene analogues. (B) The structure of the dimeric ELP–azobenzene analogues.

The synthesized ELP analogues were purified using RP-HPLC and their purity and molecular weight were confirmed using RP-UPLC-MS (Figure S1 and Table S1). These results indicated that the peptide analogue was obtained as a mixture of *trans*- and *cis*-isomers. The *cis*/*trans* ratio of each conjugate was estimated from the relative peak area of the UPLC charts to be approximately 10/90 except for 2­(F2-D)-Azb, the *cis*-/*trans*-isomers of which were not separated by UPLC under the column and solution conditions used in this study. This result is reasonable because the *trans*-conformation of azobenzene is more stable than that of the *cis*-isomer^62,63^ and in the equilibrium state before UV irradiation, *trans* is the dominant isomer. Overall, ELP–azobenzene conjugate analogues were successfully obtained with high purity via conventional and simple chemical syntheses.

### 2.2 Photoisomerization of ELP–Azobenzene Conjugate Analogues

The photoresponsive characteristics of the synthesized ELP–azobenzene conjugates were investigated. The representative absorption spectra of *trans*-azobenzene show a strong π–π* band near 320 nm and a weak n–π* band near 440 nm, whereas *cis*-azobenzene has a stronger π–π* band than that of *trans*-azobenzene near 440 nm.^59^ Upon irradiation with light of the wavelength corresponding to the π–π* band (generally UV light), the azobenzene molecule undergoes an isomerization from the *trans* to the *cis* configuration, whereas the opposite isomerization occurs upon irradiation with longer-wavelength visible light or by thermal relaxation.^64,65^ First, the absorption spectra of the ELP–azobenzene conjugates were measured in a PBS buffer solution (50 μM) to determine the *cis*-/*trans*-isomerization wavelength. Obtained absorption spectra were similar to that of typical azobenzene analogues; a strong band corresponding to π–π* transition near 330 nm and weak band corresponding to n–π* transition near 440 nm were observed (Figure 2, Table 1). Among these analogues, π–π* transition wavelength of F2-Azb-F2, F2-Azb-F2-NH_2_, and 2­(F2-D)-Azb was red-shifted and overlapped to the n–π* transition band. The substituents on the aromatic rings were reported to cause a red shift in the absorption bands of azobenzene. For example, electron-donating amino group(s) at the *ortho* or *para* positions can red-shift the spectrum.^64^ Red shift of the absorption spectra of 2­(F2-D)-Azb can be attributed to the structure of those azobenzene moieties, which possess amino groups at the *para* positions of both aromatic rings. As another case, azobenzene possessing an electron-donating group at one *para* position and an electron-withdrawing group at the other *para* position (the so-called push–pull azobenzene) can lead to further red shifts. These characteristics of F2-Azb-F2 and F2-Azb-F2-NH_2_, which correspond to push–pull azobenzene, can cause a red shift of the π–π* band in their spectra.

**Figure 2:**
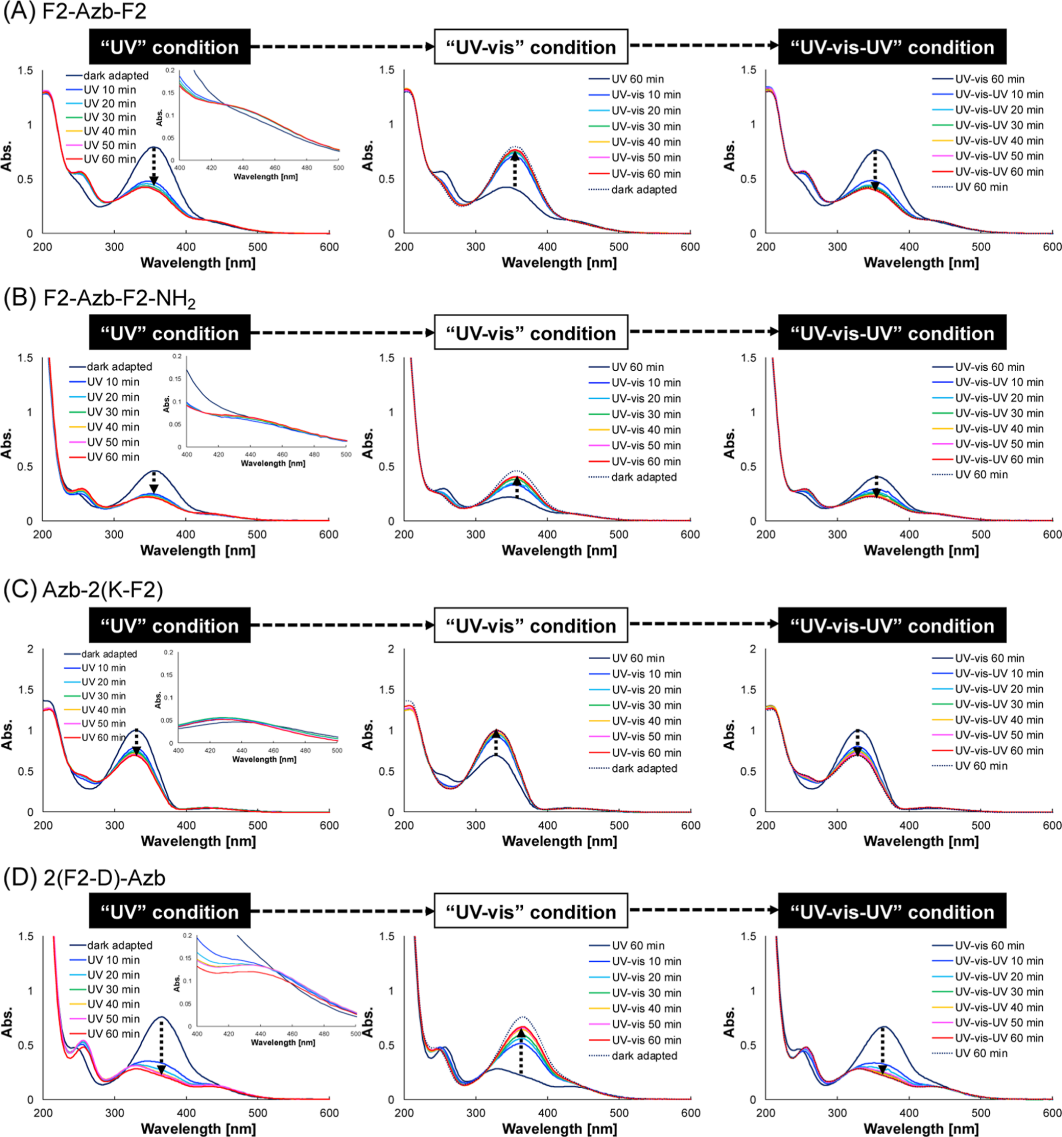
Photoisomerization of the ELP–azobenzene conjugates upon UV and visible light. Time-dependent UV–vis spectral changes of ELP–azobenzene conjugates were observed upon sequential irradiation with UV light, visible light, and UV light. Each peptide solution was prepared in a PBS solution at a concentration of 50 μM. (A) F2-Azb-F2, (B) F2-Azb-F2-NH_2_, (C) Azb-2­(K-F2), and (D) 2­(F2-D)-Azb. Inset in each left panel is an enlarged view of the wavelength range 400–500 nm. The trace of the “dark-adapted” sample is shown as a dashed black line in the UV–vis panels as a reference. The trace of the “UV 60 min” sample is shown as a dashed black line in the UV–vis–UV panels as a reference.

**Table 1:** Photo-Isomerization Properties of ELP–Azobenzene Conjugates in PBS Buffer

	peak wavelength (nm)			
peptide	π–π*	n–π*	ratio of the *cis-*/*trans*-isomer	
F2-Azb-F2	354	444	dark adapted	7.9/92.1 ± 1.3
			UV (60 min)	48.4/51.6 ± 2.1
			UV–vis (60 min)	11.6/88.4 ± 0.8
			UV–vis–UV (60 min)	44.7/55.3 ± 1.4
F2-Azb-F2-NH_2_	356	450	dark adapted	5.5/94.5 ± 1.3
			UV (60 min)	52.2/47.8 ± 0.5
			UV–vis (60 min)	17.8/82.2 ± 1.0
			UV–vis–UV (60 min)	56.1/43.9 ± 1.4
Azb-2(K-F2)	330	430	dark adapted	9.1/90.9 ± 1.1
			UV (60 min)	50.5/49.5 ± 3.6
			UV–vis (60 min)	8.6/91.4 ± 1.1
			UV–vis–UV (60 min)	53.3/46.7 ± 3.6
2(F2-D)-Azb	364	450	dark adapted	not determined
			UV (60 min)	not determined

Subsequently, PBS solutions of each peptide were continuously and alternately irradiated with UV light (corresponding to the π–π* transition) and visible light (corresponding to the n–π* transition) at 5 °C and traced its absorption spectral change every 10 min. The Xe–W lamp of a JASCO FP8500 spectrophotometer (JASCO Co.) was used as the light source for the photoisomerization of azobenzene. At first, under conditions where the dark-adapted solution of each peptide was irradiated with UV light (this condition is referred to as “UV” conditions), the time-dependent UV–vis spectra of the PBS solution of ELP–azobenzene conjugates showed a decrease in the characteristic strong π–π* transition band within 20 min, whereas the n–π* transition band at approximately 440 nm gradually increased (Figure 2). This suggested that the ELP–azobenzene conjugates changed to a *cis*-enriched state. However, after 1 h, almost no change in the spectrum was observed, even with continuous UV light irradiation, indicating that the synthesized ELP–azobenzene conjugates had reached a photostationary state. Subsequently, each peptide solution, after measurement under “UV” conditions, was irradiated with visible light to measure the time-dependent spectrum change (this condition is referred to as “UV–vis” conditions). Although the intensity of the π–π* band of each peptide increased upon irradiation with visible light, only Azb-2­(K-F2) returned to the intensity of the initial dark-adapted sample. This was considered to be due to the fact that in the other three peptides, the π–π* band was red-shifted and partially overlapped with the n–π* band, resulting in the system reaching a photostationary state with a higher *cis*-isomer content. After measurements under “UV–vis” conditions, each sample was irradiated with UV light again to confirm the reversibility of the spectral change (this condition is referred to as “UV–vis–UV” conditions). The π–π* bands of all peptides decreased to the same intensity as that observed after 60 min of UV light irradiation. These results suggest that the structural changes in the ELP–azobenzene conjugates induced by UV and visible light irradiation were reversible. However, as the π–π* transition band did not completely disappear, even after prolonged UV-light irradiation, it was estimated that the ELP–azobenzene conjugates underwent only partial conversion to the *cis*-isomer, even at the photostationary state. Therefore, the *cis*-/*trans*-isomer ratios of the analogues were investigated using UPLC analyses. It was revealed that the *cis*/*trans* ratios of the dark-adapted analogues were approximately 10/90, whereas it changed to be approximately 50/50 after “UV” conditions for 1 h (Table 1 and Figure 3). However, the *cis*- and *trans*-isomers of 2­(F2-D)-Azb could not be separated by using UPLC. ELP–azobenzene conjugates were further analyzed using UPLC following irradiation under “UV–vis” and “UV–vis–UV” conditions for 1 h. Consequently, the *cis*/*trans* ratio of these analogues was reversibly switched upon alternating irradiation with UV and visible light. A good correlation was observed between the absorbance corresponding to the π–π* of each peptide and the *cis*/*trans* ratio (Figure S2).

**Figure 3:**
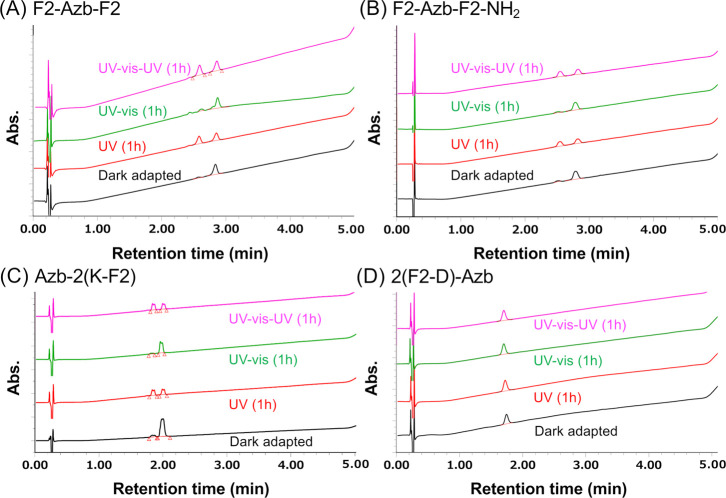
UPLC-MS analysis of the synthesized ELP–azobenzene conjugates. UPLC profiles of the ELP–azobenzene conjugates of a dark-adapted sample (black) and after irradiation under UV (red), UV–vis (green), and UV–vis–UV (magenta). (A) F2-Azb-F2, (B) F2-Azb-F2-NH_2_, (C) Azb-2­(K-F2), and (D) 2­(F2-D)-Azb. As shown in (D), the *cis*- and *trans*-isomers of 2­(F2-D)-Azb could not be divided.

The thermal stability of the ELP–azobenzene conjugates was also investigated by using UV–vis spectroscopy. After measurements under “UV–vis–UV” conditions, each peptide solution was incubated in the dark, and spectral changes were monitored over time (Figure 4). The intensity of the π–π* band of the conjugates, except for Azb-2­(K-F2), gradually increased even when incubated at 5 °C, reaching an intensity comparable to that of the dark-adapted samples within 20 h. Thus, it was suggested that thermal isomerization of these analogues from the *cis*-isomers to the *trans*-isomers readily occurred in these analogues. In contrast, Azb-2­(K-F2) remained in a *cis*-enriched state even after standing at 37 °C for 12 h under light-protected conditions (Figure 4C). Furthermore, Azb-2­(K-F2) retained its *cis*-isomer-enriched state even after 3 days (Figure S3). These findings indicate that the photoswitching behavior of this peptide was more readily controllable than that of the other analogues.

**Figure 4:**
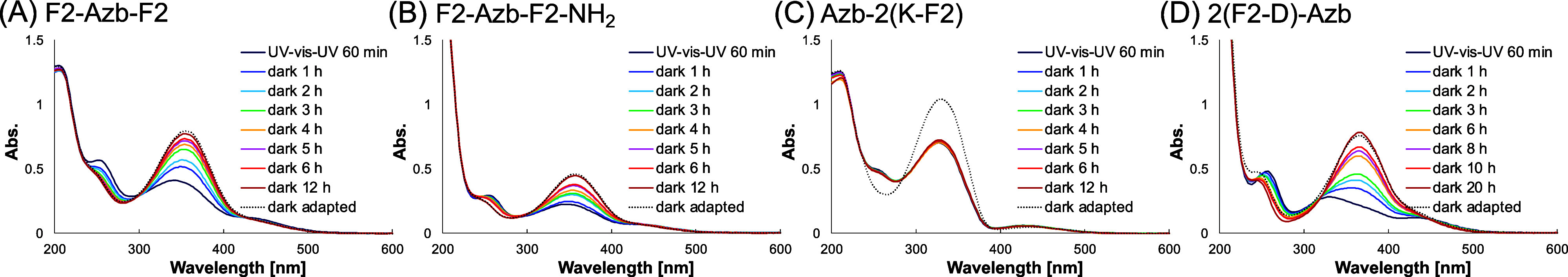
Thermal isomerization of the ELP–azobenzene conjugates under dark conditions after UV light irradiation. Time-dependent UV–vis spectral changes of ELP–azobenzene conjugates under dark conditions following irradiation with UV light. Each peptide solution was prepared in PBS at a concentration of 50 μM. (A) F2-Azb-F2 (at 5 °C), (B) F2-Azb-F2-NH_2_ (at 5 °C), (C) Azb-2­(K-F2) (at 37 °C), and (D) 2­(F2-D)-Azb (at 5 °C). Dotted line: UV–vis spectra of the dark-adapted solutions (i.e., before UV-light irradiation) of each conjugate.

Additionally, the isomerization of Azb-2­(K-F2) to a *cis*-enriched state was induced by irradiation with UV-A light (365 nm), which is a commercially available light source (Figure S4). However, because the wavelength of UV-A light is longer than the absorption maximum of the π–π* band of this peptide, irradiation at 365 nm resulted in a photostationary state with a lower *cis*-isomers ratio than that achieved with UV light (Figure S4A). On the other hand, when an LED light with a higher irradiance than that used for spectrum measurements was used as the light source, Azb-2­(K-F2) reached a photostationary state in approximately 5 min (Figure S4B). Overall, based on the experimental results, Azb-2­(K-F2) appears to be a promising easy-to-handle peptide with both temperature and photoresponsiveness.

### 2.3 Turbidity Measurement of ELP–Azobenzene Conjugate Analogues

The ability of the ELP–azobenzene conjugates to self-assemble was investigated by using turbidity measurements. In addition, we evaluated whether the photoisomerization of the azobenzene moieties induced differences in the aggregation ability of these analogues. Each peptide analogue was dissolved in PBS at different concentrations according to its water solubility. To quantitatively evaluate the aggregation ability, the transition temperature (*T*_t_) of the peptides was calculated from the change in optical density at a wavelength of 600 nm (OD_600_) associated with the increasing temperature (Table 2 and Figure 5). It was revealed that the *T*_t_ value of the ELP–azobenzene conjugates increased compared to that of the dark-adapted solutions after irradiation under “UV” conditions for 1 h. This result indicates that the aggregation ability of the *cis*-isomer of the ELP–azobenzene conjugates is weaker than that of the *trans*-isomer. This could be attributed to changes in the hydrophilicity of the azobenzene moiety associated with photoisomerization. In other words, azobenzene undergoes a configurational switch from the *trans*- to *cis*-form upon UV-light irradiation, accompanied by a change from a hydrophobic to a more hydrophilic state.^58,61^ As the phase separation of short ELP analogues can be significantly affected by local structural changes,^66^ it was considered that the aggregation ability of the ELP–azobenzene conjugates changed due to the change in the hydrophilicity accompanying the photoisomerization of the azobenzene moiety. Among the analogues, 2­(F2-D)-Azb showed the largest change in the phase separation behavior; this analogue did not exhibit phase separation at a concentration of 0.66 mM (1.5 mg/mL) after irradiation under “UV” conditions.

**Table 2:** Phase Transition Temperature Values of the ELP–Azobenzene Conjugates^a^

peptide	concentration	*T*_t,dark_ (°C)	*T*_t,UV_ (°C)	*T*_t,UV–vis_ (°C)	*T*_t,UV–vis–UV_ (°C)
F2-Azb-F2	100 μM (0.21 mg/mL)	18.63 ± 0.99	35.61 ± 0.56	20.91 ± 0.08	33.36 ± 0.55
F2-Azb-F2-NH_2_	50 μM (0.11 mg/mL)	14.88 ± 0.56	32.60 ± 1.14	19.18 ± 0.43	31.53 ± 1.66
Azb-2(K-F2)	1.27 mM (3.00 mg/mL)	32.09 ± 0.14	35.08 ± 0.30	32.55 ± 0.67	35.94 ± 1.15
2(F2-D)-Azb	0.66 mM (1.50 mg/mL)	20.63 ± 0.41	not determined	25.76 ± 0.58	not determined

a The phase transition temperatures of ELP–azobenzene conjugates before and after UV-light irradiation. Each peptide was dissolved in PBS (pH 7.4). *T*_t,dark_: transition temperature of the dark-adapted solution, *T*_t,UV_: transition temperature after irradiation under “UV” conditions, *T*_t,UV–vis_: transition temperature after irradiation under “UV–vis” conditions, *T*_t,UV–vis–UV_: transition temperature after irradiation under “UV–vis–UV” conditions. Each assay was repeated three times with different peptide solutions that had been separately prepared.

**Figure 5:**
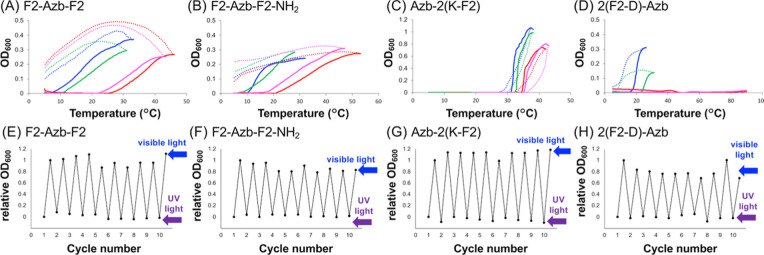
Turbidity measurements of ELP–azobenzene conjugates. Turbidity changes of the ELP–azobenzene conjugates in PBS solutions during heating (solid lines) and cooling (dashed lines). (A) F2-Azb-F2 (100 μM), (B) F2-Azb-F2-NH_2_ (50 μM), (C) Azb-2­(K-F2) (1.27 mM), and (D) 2­(F2-D)-Azb (0.66 mM). Turbidity measurements were carried out for the dark-adapted solutions (blue lines) and after irradiation with “UV” conditions (red lines), “UV–vis” conditions (green lines), and “UV–vis–UV” conditions (magenta lines). (E)–(H) shows the reversibility of the light-mediated transition (relative optical density at 600 nm) over 10 cycles of 10 min irradiation of (E) F2-Azb-F2 (at 20 °C), (F) F2-Azb-F2-NH_2_ (at 20 °C), (G) Azb-2­(K-F2) (at 37 °C), and (H) 2­(F2-D)-Azb (at 25 °C).

Subsequently, turbidity measurements were conducted after irradiation under “UV–vis” and “UV–vis–UV” conditions for 1 h to examine the reversibility of the changes in phase separation characteristics induced by light irradiation. Although the *T*_t_ values of these peptides decreased after irradiation under “UV–vis” conditions, the *T*_t_ values were higher than those of the dark-adapted samples, except for Azb-2­(K-F2). In contrast, the *T*_t_ values of the analogues after irradiation under “UV–vis–UV” conditions were similar to those observed after irradiation under “UV” conditions. These results were consistent with the spectral measurement data. As shown in revised Figure S5, a good correlation was observed between ratio of the *cis*-isomer ratio of the ELP–azobenzene conjugate and the *T*_t_ values. From this correlation, it was considered that the *T*_t_ could be estimated by determining the ratio of the *cis*-isomer in the solution of each analogue. Furthermore, the reversibility of the light-induced phase separation of each analogue was examined by measuring the OD_600_ changes during 10 successive isothermal irradiation cycles. The change in OD_600_ with light irradiation was almost the same for 10 consecutive cycles (Figure 5E–H), indicating that the effect of isomerization on phase separation was reproducible across multiple irradiation cycles.

The phase separation ability of the dimeric ELP–azobenzene conjugate analogues, which were more water-soluble than the linear analogues, was examined not only in PBS but also in phosphate buffer solutions. Azb-2­(K-F2) and 2­(F2-D)-Azb underwent thermoresponsive phase separation in a neutral phosphate buffer solution (pH 7.4) as well as in PBS, whereas these peptides did not show phase separation in acidic (pH 2.1) or basic (pH 11.5) phosphate buffer solutions (Table S2 and Figure S6). Therefore, it was hypothesized that dimeric peptides might exist as zwitterions in neutral aqueous solutions and form intermolecular electrostatic interactions that could neutralize the charges of the peptide molecules, thereby enhancing their aggregation ability. This hypothesis was supported by the turbidity measurement of Azb-2­(F2), an analogue of Azb-2­(K-F2) lacking lysine residues: Azb-2­(F2) did not exhibit phase separation even at 2.38 mM (5.00 mg/mL) in PBS (Figure S7), presumably due to the deprotonation of the carboxyl groups and the absence of counter ammonium ions.

The *T*_t_ of the ELP–azobenzene conjugates synthesized in this study changed depending on the ratio of the *cis*-isomer. In particular, the *T*_t_ of the hydrophobic linear analogues increased significantly with a higher *cis*-isomer ratio, whereas the change in *T*_t_ for Azb-2­(K-F2), which contains hydrophilic Lys residues, was minimal. These results suggest that the effect of the *cis*-isomer ratio on *T*_t_ depends on the hydrophobicity of the peptide. Meanwhile, 2­(F2-D)-Azb did not show a phase transition in the *cis*-isomer state, possibly because the peptide concentration required to induce the transition under *cis*-rich conditions exceeded the concentration used in this study. Further investigation is needed to clarify how the peptide concentration and the *cis*-/*trans*-isomer ratio affect the phase transition behavior.

### 2.4 Size Distribution Analysis of the ELP–Azobenzene Conjugate Analogues

The temperature- and photoresponsive behaviors of the ELP–azobenzene conjugates were further investigated by size distribution measurements using dynamic light scattering (DLS) (Figure 6). The size distribution histograms of each conjugate were obtained from the DLS autocorrelation curves (Figure S8) using a cumulant fit performed with Zetasizer software. As a result of DLS measurements of dark-adapted F2-Azb-F2, this analogue formed submicron-ordered aggregates (approximately 200 nm) at 5 °C and gradually increased to nearly 1 μm in the hydrodynamic diameter associated with an increasing temperature to 25 °C. However, after irradiation under “UV” conditions, F2-Azb-F2 retained the state of submicron aggregates at 25 °C and required 35–45 °C to form micrometer-sized particles. This was consistent with the result of the turbidity measurements in which the *T*_t_ value of F2-Azb-F2 was 18.6 and 35.6 °C before and after irradiation, respectively. F2-Azb-F2-NH_2_ exhibited particle size distribution profiles similar to those of F2-Azb-F2. The dark-adapted dimeric ELP–azobenzene conjugates (Azb-2­(K-F2) and 2­(F2-D)-Azb) were in equilibrium between nanometer-sized and submicrometer-sized particles at low temperatures, followed by maturation to micrometer-sized particles with the increasing temperature. In addition, the temperatures at which the particle size of Azb-2­(K-F2) changed were increased by UV-light irradiation, corresponding to the *T*_t_ changes in the turbidity measurements. Conversely, 2­(F2-D)-Azb after irradiation of UV for 1 h, which did not self-assemble in the turbidity measurement, remained in equilibrium between the monomeric (nanometer-ordered particles) and minute aggregate states (submicrometer-ordered particles) regardless of temperature changes and did not form micrometer-ordered particles. The similar result was obtained by DLS measurement of Azb-2­(F2), which did not exhibit self-assembly even in the dark-adapted solution (Figure S9). Among the ELP–azobenzene conjugates, changes in the Azb-2­(K-F2) particle size were induced by subtle temperature differences. Specifically, the particle size distribution changed between 31 and 34 °C before irradiation, whereas it changed between 34 and 37 °C after 330 nm irradiation for 1 h. This suggests that the phase separation of Azb-2­(K-F2) can be precisely controlled by both temperature and light irradiation.

**Figure 6:**
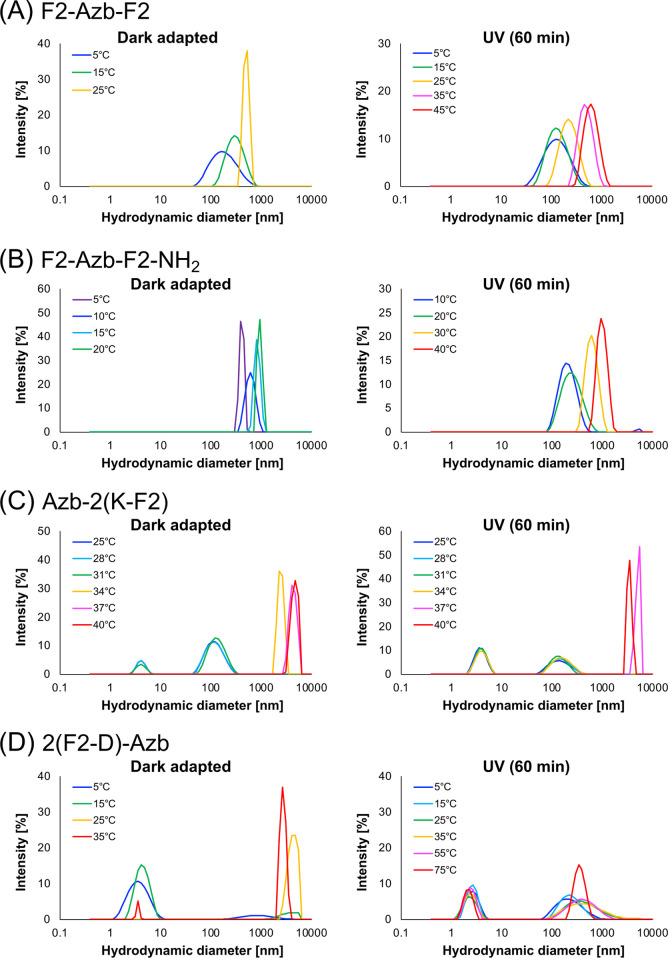
Effect of photoisomerization on the particle size distribution of ELP–azobenzene conjugates. DLS measurements were carried out before and after irradiation of UV light, corresponding to each π–π* transition band for 1 h. (A) F2-Azb-F2 (100 μM in PBS), (B) F2-Azb-F2-NH_2_ (50 μM in PBS), (C) Azb-2­(K-F2) (1.27 mM in PBS), and (D) 2­(F2-D)-Azb (0.66 mM in PBS).

### 2.5 Microscopic Study of the ELP–Azobenzene Conjugate Analogues

To investigate the morphological properties of the ELP–azobenzene conjugates, a PBS solution of Azb-2­(K-F2) was observed by using optical microscopy at different temperatures under light-protected conditions. The Azb-2­(K-F2) solution was almost homogeneous at 25 °C (Figure 7A). However, spherical coacervates of the peptide emerged at 35 °C and then rapidly matured into larger particles with diameters of several micrometers by increasing the temperature to 37 °C (Figure 7B,C). These particles stably exist under dark conditions (Figure 7D). Notably, the particles disappeared after irradiation with UV-A light (365 nm) for 5 min (Figure 7E), suggesting that Azb-2­(K-F2) transitioned to a *cis*-enriched state upon light irradiation, resulting in a decreased aggregation ability and redissolution of the formed particles. Subsequently, after exposure to room light for 5 min, spherical aggregates reappeared (Figure 7F), implying that the peptide reverted to a *trans*-enriched state under visible light. In addition, 2­(F2-D)-Azb showed a similar trend in PBS (Figure S10). These results demonstrate that the formation of ELP–azobenzene conjugate aggregates can be controlled by light irradiation at different wavelengths.

**Figure 7:**
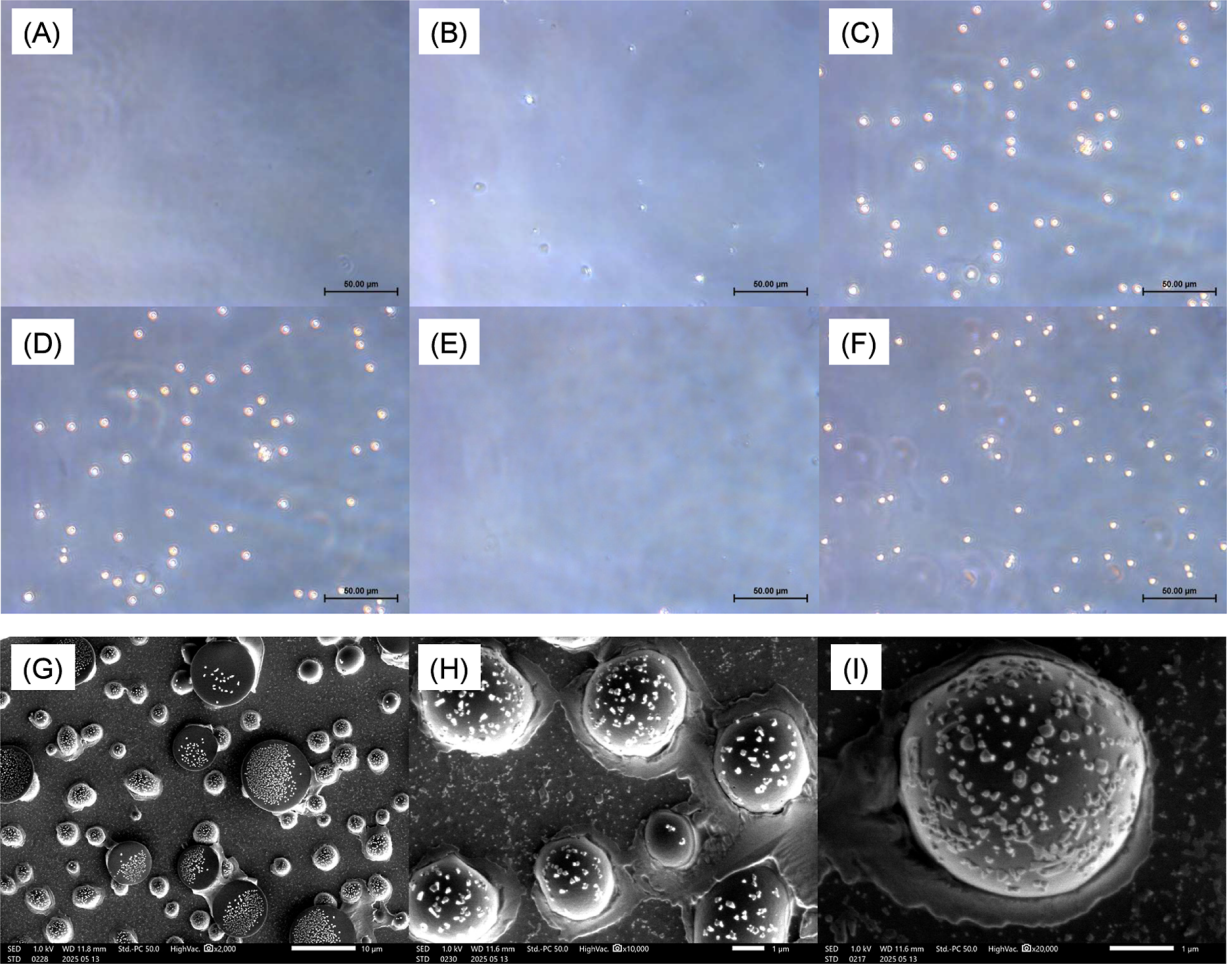
Optical and electron microscopy images of Azb-2­(K-F2) under various thermal and light conditions. Optical microscopic images of the PBS solution of Azb-2­(K-F2) (3 mg/mL) at (A) 25 °C, (B) 35 °C, (C) 37 °C, and (D) after incubation under 37 °C for 1 h. (E) After UV-A light irradiation (365 nm) for 5 min at 37 °C. (F) After incubation under room light for 5 min at 37 °C subsequent to incubation (E). The magnification of all the images is 40×. Scale bars are 50 μm. Scanning electron microscopic images of Azb-2­(K-F2) incubated at 37 °C are shown in (G)–(I). The magnification is 2000× for (G), 10,000× for (H), and 20,000 x for (I). Scale bars represent 10 μm in (G), and 1 μm in (H) and (I).

Furthermore, the morphologies of the self-assemblies of Azb-2­(K-F2) were determined using scanning electron microscopy. The peptide sample was prepared by incubating a PBS solution of Azb-2­(K-F2) at 37 °C. As a result, spherical particles with a diameter of 5–10 μm, formed by aggregation of Azb-2­(K-F2), were clearly observed (Figure 7G–I). Additionally, numerous amorphous particles (approximately 100 nm in size), which may possibly be microaggregates of the peptide formed below the transition temperature, were incorporated on the surface of the spherical particles. Therefore, as shown by the DLS results, Azb-2­(K-F2) is considered to form submicrometer-sized particles below the *T*_t_ and mature into micrometer-sized particles with the increasing temperature. It was also confirmed that the formation of micrometer-sized particles was suppressed by UV-A light irradiation during incubation at 37 °C (Figure S11).

Azb-2­(K-F2) was further studied to determine whether the chemical release from the peptide aggregates could be controlled by light irradiation. Azb-2­(K-F2) and Rhodamine B (a red fluorescent dye) were heated in PBS to form coacervates, and a stable pellet of the aggregate was obtained by centrifugation. This pellet was composed of Azb-2­(K-F2) coacervates containing rhodamine B with diameters of several micrometers, as shown in Figure 7. The structure of the obtained sample was observed over time by using optical and fluorescence microscopy under irradiation with monochromatic light at a wavelength of 365 nm (Figure 8). Before light irradiation, the peptide aggregate containing Rhodamine B was observed as a red-colored amorphous structure with a size of approximately 600–700 μm. When irradiated with UV-A light, spherical particles (several micrometers in diameter) leaked from the surface of the aggregates and the red dye diffused accordingly. This dissociation process was considered to be the reverse of the stepwise phase separation in which ELP formed submicron-ordered aggregates and then matured to larger aggregates with diameters of several micrometers.^67^ As a result of further continued light irradiation, the structure of the aggregates completely collapsed, and the fluorescent dye diffused over a wide area. In contrast, the peptide aggregate containing Rhodamine B was stable at 37 °C in the absence of UV-A light (Figure S12). These characteristics of the ELP–azobenzene-conjugated analogues indicate that drug release from the particles of these peptide analogues can be controlled by light irradiation, suggesting their potential application as photoresponsive drug carriers.

**Figure 8:**
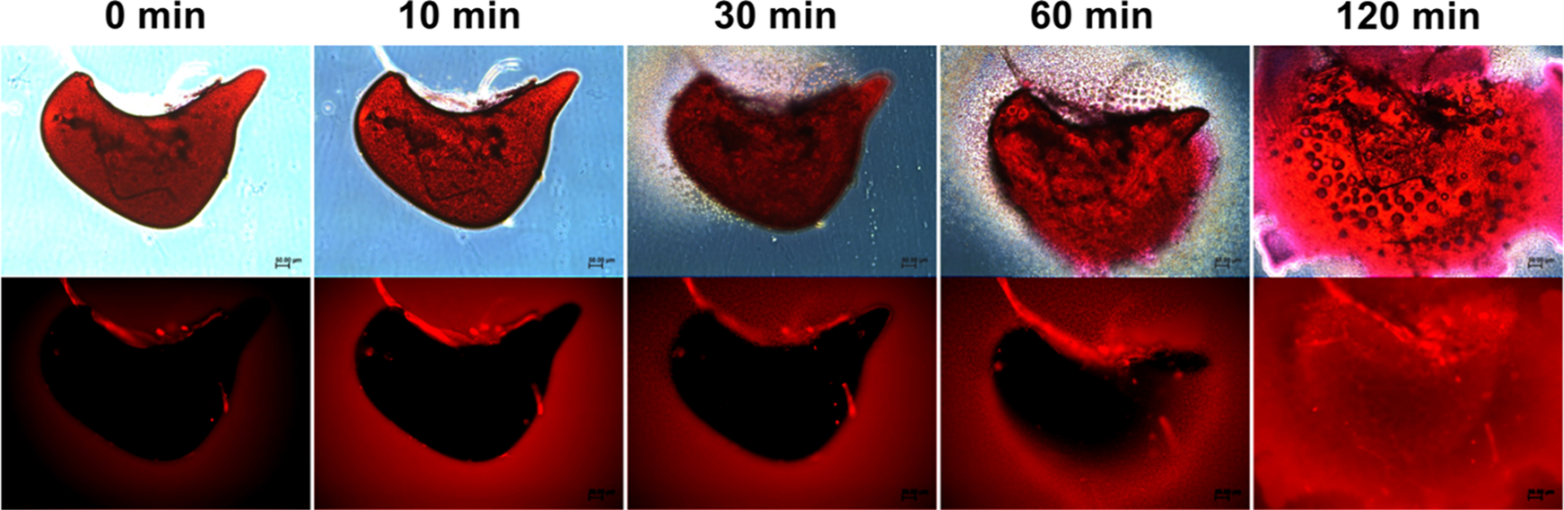
Microscopy images of Azb-2­(K-F2) aggregates containing Rhodamine B. Optical (upper row) and fluorescence (lower row) microscopic images of Azb-2­(K-F2) aggregates containing Rhodamine B in a PBS solution. Time-course observation of the prepared sample was carried out at 37 °C under irradiation with 365 nm of monochromatic light. Irradiation times are indicated at the top of the figure. The magnification of all the images is 10×. Scale bars indicate 50 μm.

### 2.6 Computational Studies of ELP–Azobenzene Conjugates

Various studies have been conducted to elucidate the structural properties involved in the phase-separation mechanism of the ELPs. Several studies have demonstrated that the phase separation of ELPs has been considered to be related to the formation of β-turn structures by four consecutive amino acid residues flanking the Pro–Gly motif (in this study, Phe–Pro–Gly–Val) in response to increasing temperatures.^68^ To evaluate whether the structural change of ELP with the temperature change was affected by the conjugation with azobenzene, MD simulations were performed for F2-Azb-F2. MD simulations of 30 ns were performed using a molecular model of the *trans*- and *cis*-isomer of F2-Azb-F2 at 278 and 303 K, respectively. Trajectory data from the final 20 ns were analyzed to assess the conformational behavior of F2-Azb-F2. The formation of type-II β-turns involving the Phe–Pro–Gly–Val sequence was assessed using tow criteria: (i) the distances between the oxygen atom of the Phe and the amide proton of the Val (>0.25 nm), and (ii) the dihedral angles (Φ and Ψ) of the tandem Pro–Gly residues within each pentapeptide unit. Especially, we investigated whether the PG residues in each repeat satisfied the combination of dihedral angles for type-II β-turns, where Φ_2_ = 60°, Ψ_2_ = 120°, Φ_3_ = 80°, and Ψ_3_ = 0° with the error range defined as ±40° (Figure 9, area displayed by the dotted circle).^69^ As a result, the frequency of the formation of type-II β-turns clearly increased at 303 K for both of the isomers compared to that at 278 K. These findings indicated that the structural changes in the peptide components with temperature changes were not affected by conjugation with azobenzene. Therefore, the differences in the aggregation behavior of the *cis* and *trans* isomers of the ELP–azobenzene conjugates are likely attributable to changes in the hydrophilicity of the azobenzene moiety upon photoisomerization rather than alterations in the three-dimensional structure of ELP itself upon photoisomerization.

**Figure 9:**
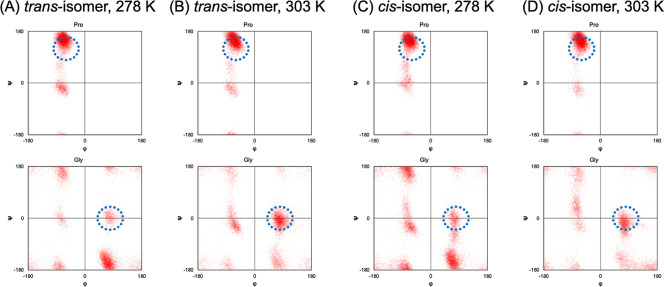
Dihedral angles were adapted by tandem Pro and Gly residues in each pentapeptide repeat of F2-Azb-F2. (A) *Trans*-isomer at 278 K, (B) *Trans*-isomer at 303 K, (C) *Cis*-isomer at 278 K, and (D) *Cis*-isomer at 303 K. Dotted circles in each panel indicate the range of dihedral angles (Φ and Ψ) typically found in type-II β-turns.

## 3 Conclusions

In this study, temperature- and photoresponsive self-assembling peptide analogues were developed by conjugating short ELPs and azobenzene derivatives. The synthesized ELP–azobenzene conjugate exhibited reversible spectral changes upon UV or visible-light irradiation. UPLC analyses revealed that the *trans*/*cis* ratio of ELP–azobenzene conjugates after 1 h of UV irradiation was approximately 50/50, indicating that these ELP–azobenzene conjugates were not completely converted to the *cis*-isomer. Turbidity and DLS measurements revealed that the *trans*-isomers of the ELP–azobenzene conjugates exhibited a stronger aggregation ability than the *cis*-isomers. This was attributed to the change in the hydrophilicity of the azobenzene moiety upon photoisomerization from the *trans*- to *cis*-form, which affects the water solubility of the ELP–azobenzene conjugates. This hypothesis was supported by computational studies showing that the temperature-dependent structural changes in the peptide components were similar between the two isomers. Additionally, microscopic observations demonstrated that aggregate formation and compound release from ELP–azobenzene aggregates could be controlled by light irradiation, suggesting the potential of these peptides as photoresponsive drug carriers.

Recently, the development of protein-based biomaterials that combine photoresponsive molecules with ELPs has been reported. For example, Amiram et al. have demonstrated the precise incorporation of photoresponsive molecule-bearing unnatural amino acids into a single ELP at the genetic level by using a highly efficient orthogonal aminoacyl-tRNA synthetase.^57,58^ This technique enables the creation of light-responsive protein-based biomaterials both in vitro and within bacterial cells, providing powerful strategies for regulating the functions of biomolecules and understanding complex cellular behaviors. In contrast, the ELP–azobenzene conjugates developed in this study were small molecules that could be readily synthesized through chemical reactions similar to peptide bond formation. This allows for the flexible addition or substitution of amino acid residues, as required. In this regard, a liquid-phase synthesis method using soluble hydrophobic tags developed by Chiba et al. has recently attracted attention as a suitable technique for large-scale peptide synthesis.^70–72^ We have previously reported the application of this method for the facile synthesis of ELPs.^73^ Therefore, large-scale production of ELP–azobenzene conjugates is also expected to be feasible, which may facilitate the development of photo- and temperature-responsive biomaterials that can be synthesized and applied in bulk under in vitro conditions. Furthermore, the discovery of an analogue that can form stable photo- and temperature-responsive aggregates with a single component, even under physiologically relevant conditions, could enable applications in light-controlled drug delivery systems. While the ELP–azobenzene conjugates developed in this study offer advantages in terms of production methodology, further improvements in functionality, such as a more defined switching behavior and enhanced controllability of responsiveness to light and temperature, are needed. We are currently conducting research to achieve these improvements.

## 4 Materials and Methods

### 4.1 Chemicals

Fmoc-amino acids, Fmoc-NH-SAL-MBHA resin (100–200 mesh), Fmoc-Gly-Wang resin (100–200 mesh), *N*,*N*-diisopropyl-ethylamine (DIPEA), 2-(1*H*-benzotriazole-1-yl)-1,1,3,3-tetramethyl uronium hexafluorophosphate (HBTU), ethyl cyano­(hydroxyimino)­acetate (OxymaPure), piperidine, 1-((dimethylamino)­(dimethyliminio)­methyl)-1*H*-[1,2,3]­triazolo-[4,5-*b*]­pyridine 3-oxide hexafluorophosphate (HATU), 3*H*-[1,2,3]­triazolo­[4,5-*b*]­pyridin-3-ol (HOAt), trifluoroacetic acid (TFA), and dichloromethane were purchased from Watanabe Chemical Industries Ltd. (Hiroshima, Japan). Triisopropylsilane (TIS), azobenzene-4,4′-dicarbonyl dichloride, and Rhodamine B were purchased from Tokyo Chemical Industries Co., Ltd. (Tokyo, Japan). *N*,*N*-Dimethylformamide (DMF) and ethyl acetate were purchased from Kanto Chemical Co., Ltd. (Tokyo, Japan). Tetrahydrofuran (THF) was purchased from Nacalai Tesque, Inc. (Kyoto, Japan). 4-((4-Aminopheyl)­diazenyl)­benzoic acid was purchased from BLD Pharmatech Ltd. (Shanghai, China). Water for experiment was purified by Milli-Q Integral 3 (Merck Millipore, Darmstadt, Germany). Other solvents and reagents were also obtained from commercial suppliers and used without further purification.

### 4.2 Peptide Synthesis

Elastin-like peptide analogues were synthesized by the conventional solid-phase method using the Fmoc strategy. H-(FPGVG)_2_–OH and its analogues were synthesized using a CSBIO II peptide synthesizer (Menlo Park, CA) using HBTU and OxymaPure as coupling reagents. The peptides were cleaved from the resin in a cleaving cocktail containing 95% TFA/2.5% TIS/2.5% H_2_O. The synthesized peptide analogues were prepurified using a Sep-Pak Vac 35 cc C18 cartridge (Waters Co., Milford, MA). Further purification was performed using a reversed-phase (RP)-HPLC system (JASCO PU-2089 equipped with UV-2075 or JASCO PU-4180 equipped with UV-4075, JASCO, Tokyo, Japan) equipped with a C8 column (COSMOSIL 5C8-AR-300 packed column, 20 mm I. D. × 150 mm, 5 μm, 300 Å, Nacalai Tesque Inc.). The purity and molecular weights of the peptides were confirmed using ACQUITY ultraperformance liquid chromatography (UPLC) H-Class (Waters Co.) equipped with a QDa MS detector and an ACQUITY UPLC BEH C-18 column (100 mm, Waters Co.). Further detailed protocols of the peptide synthesis and purification methods are shown in the Supporting Information.

### 4.3 Synthesis of ELP–Azobenzene Conjugates

ELP–azobenzene conjugate analogues were synthesized by conjugating short ELP and azobenzene analogues. The chemical structures of the ELP–azobenzene conjugate analogues synthesized in this study are summarized in Figure 1 and Table S1. Further detailed protocols for the peptide synthesis and purification methods are provided in the Supporting Information.

### 4.4 Photoisomerization of ELP–Azobenzene Conjugates

The synthesized ELP–azobenzene conjugates were dissolved in PBS buffer (137 mM NaCl, 2.68 mM KCl, 10 mM Na_2_HPO_4_, and 2 mM KH_2_PO_4_, pH 7.4) to prepare 50 μM solutions. The time-dependent UV–vis spectra of each conjugate were recorded using a JASCO V-660 spectral photometer (JASCO Co., Tokyo) in a quartz cell (optical path length: 10 mm, volume: 400 μL). The light source for both UV and visible light in this study is a 150 W xenon arc lamp of a JASCO FP8500 spectrophotometer, which is capable of emitting light in the wavelength range of 200 to 750 nm. Initially, the absorption spectra of the dark-adapted solution of the ELP–azobenzene conjugate were measured to determine the irradiation wavelength corresponding to its π–π* band. Subsequently, the ELP–azobenzene conjugate solution was irradiated with light corresponding to the π–π* band for 60 min and the absorption spectra were recorded every 10 min. Next, the peptide solution was irradiated with visible light corresponding to the n–π* band for 60 min and the absorption spectra were recorded every 10 min. The peptide solution was then irradiated again with UV light for 60 min and the absorption spectra were recorded every 10 min. Finally, the peptide solution was incubated in the dark conditions and the absorption spectra were recorded every 1 h. The *cis*/*trans* ratio of the ELP–azobenzene conjugates was determined from the relative peak area obtained from the UPLC analyses.

### 4.5 Turbidity Measurement

The temperature-dependent self-assembly property of synthesized peptides was evaluated by using a JASCO V-660 spectral photometer (JASCO Co.). Each peptide was dissolved in PBS buffer or phosphate buffer solutions at various concentrations. Three types of phosphate buffers with the same ionic strength (μ = 0.100) but different pH (pH 7.4, 2.1, and 11.5) were prepared: phosphate buffer (pH 7.4, 29.4 mM Na_2_HPO_4_, 11.7 mM NaH_2_PO_4_), phosphate buffer (pH 2.1, 10.0 mM NaH_2_PO_4_, 10.5 mM phosphoric acid), and phosphate buffer (pH 11.5, 9.58 mM Na_3_PO_4_, 14.2 mM Na_2_HPO_4_). Turbidity was measured at 600 nm with increasing or decreasing temperature at a rate of 0.5 °C/min from 5 °C. The measurements were performed for the dark-adapted solution and after successive irradiation with UV light, visible light, and UV light. Each assay was repeated three times with different peptide solutions that were prepared separately. The phase separation property was described by the phase transition temperature (*T*_t_), which is the temperature at which the turbidity of the solution reaches half its maximum value.

### 4.6 The Particle Size Measurement by DLS

The particle size distribution in ELP–azobenzene conjugate solutions was analyzed by DLS measurement on a Zetasizer nano ZS (Malvern Instruments Ltd., Worcestershire, UK.). The peptide samples were dissolved in PBS buffer at various concentrations. The DLS measurement was conducted with an increasing temperature below and above the *T*_t_. The measurement duration was selected automatically. A data set of protein (refractive index = 1.450, absorption = 0.001) as the material and water (refractive index = 1.330, viscosity = 0.8872) was used for analysis. Attenuation was selected automatically. Each sample solution was measured at least three times.

### 4.7 Optical Microscopic Study

The morphology of the coacervates of the ELP–azobenzene conjugate Azb-2­(K-F2) was observed by optical microscopy. The light field observation was performed using a Leica DM IL LED (Leica Microsystems CMS, Wetzlar, Germany) equipped with HI PLAN 40× (Leica Microsystems CMS), HC PLAN 10× (Leica Microsystems CMS), and a Leica EL6000 external light source (Leica Microsystems CMS). Azb-2­(K-F2) was dissolved in PBS buffer at a concentration of 3 mg/mL. Sample imaging was performed at 25, 35, and 37 °C by using a Thermo Plate TP-CHSQM (Tokai Hit Co., Ltd., Shizuoka, Japan). Then, the sample was observed after irradiation with 365 nm monochromic light for 15 min using a Mounted LED M365L3 (Thorlabs Japan Inc., Tokyo, Japan).

A time-course observation of Azb-2­(K-F2) aggregates in the presence of a fluorescent dye was also performed. To the Azb-2­(K-F2) solution (in phosphate buffer, pH 7.4, 3 mg/mL) was added Rhodamine B at a final concentration of 50 μM. The resulting solution was incubated at 37 °C for 4 h. Then, the solution was centrifuged for 20 s (6200 rpm) to obtain an aggregate of the peptide as a red pellet. The obtained peptide aggregate was washed with PBS buffer (37 °C, 50 μL, three times) and was used to make a prepared slide. Time-dependent observation of the prepared sample was carried out at 37 °C under irradiation with 365 nm monochromatic light.

### 4.8 Scanning Electron Microscopic Study

PBS solution of 1.27 mM of Azb-2­(K-F2) was dropped onto a cover glass and left at 37 °C for air drying under dark conditions or irradiation with UV-A light (wavelength of 365 nm). The prepared samples were osmium-coated with an HPC-1SW osmium plasma coater (Vacuum Device Co., Ibaraki, Japan) and examined using a JSM-IT700HR InTouchScope (JEOL, Tokyo, Japan) at an operating voltage of 1.0 kV.

### 4.9 Computational Studies of ELP–Azobenzene Conjugates

The structural changes associated with the phase separation ability of ELP–azobenzene conjugates were investigated by using molecular dynamics (MD) simulations. All calculations were performed using a DELL PRECISION T3610 workstation (Dell Inc., Round Rock, TX, USA). MD simulations were performed using GROMACS 2019 software with an Amber ff99SB-ILDN force field. In this study, the structural dynamics of F2-Azb-F2 was analyzed. The TIP3P explicit solvent model was used to analyze the interactions between the peptide and water molecules. Initial conformations were generated using Discovery Studio 4.5 (Dassault Systemes BIOVIA, San Diego, CA, USA). The structural properties of *trans*- and *cis*-isomers of F2-Azb-F2 were then calculated via MD simulations at 278 and 303 K, respectively. The detailed calculation protocols are described in the Supporting Information.

## Supplementary Material



## Notes

Detailed procedure for synthesis of ELP analogues, synthesis of ELP–azobenzene conjugates, purification of the peptides, MD simulations, ELP analogue synthesized in this study, phase transition temperature values of ELP–azobenzene conjugates in phosphate buffer solutions, UPLC–MS analysis of the synthesized ELP–azobenzene conjugates, correlation between the absorbance corresponding to the π–π* of each peptide and ratio of the *cis*-isomer, thermal stability of Azb-2­(K-F2) under dark conditions, photoisomerization of Azb-2­(K-F2) upon UV-A light irradiation, correlation between the ratio of the *cis*-isomer and the phase transition temperature (*T*_t_), turbidity measurements of the ELP–azobenzene conjugates in phosphate buffer solutions, structure and turbidity measurements of Azb-2­(F2), DLS measurements and autocorrelation curves, particle size distribution of Azb-2­(F2), optical microscopy images of 2­(F2-D)-Azb in PBS solution, scanning electron microscopy images of Azb-2­(K-F2) prepared with irradiation of UV-A light, and microscopy images of Azb-2­(K-F2) aggregates containing Rhodamine B under dark conditions (PDF)
