# Supplementary material for: Azobenzene-Modified Temperature-Responsive Short Elastin-like Peptides for Photo-Controlled Phase Transition
Source: ACS Synth Biol. 2025 Jul 24;14(8):2999–3012. doi: 10.1021/acssynbio.4c00889 (PMC12363518; doi:10.1021/acssynbio.4c00889)
Supplement: Supplementary file 1 [file sb4c00889_si_001.pdf]

## Supplementary information

# Azobenzene-modified temperature-responsive short elastin-like peptides for photocontrolled phase transition


**AUTHOR NAMES**

*Keitaro Suyama,[1,2*] Elissa Ngoc Mai,[2] Iori Maeda,[3] and Takeru Nose[1,2*]*

**AUTHOR ADDRESS**

*[1] Faculty of Arts and Science, Kyushu University, Fukuoka, 819-0395, Japan. [2] Department of Chemistry, Faculty and Graduate School of Science, Kyushu University, Fukuoka, 819-0395, Japan. [3] Department of Physics and Information Technology, Kyushu Institute of Technology, Iizuka, Fukuoka 820-8502, Japan.*

Manuscript Correspondence:
Prof. Takeru Nose
Tel: +81-92-802-6025
Fax: +81-92-802-6025
e-mail: nose@artsci.kyushu-u.ac.jp

Assistant Prof. Keitaro Suyama
Tel: +81-92-802-5849
e-mail: suyama@artsci.kyushu-u.ac.jp

**Materials and Methods** ..........................................................................(S3–S6)
Synthesis of elastin-like peptide analogs.
Synthesis of ELP-azobenzene conjugates.
Purification of the peptides.
Molecular Dynamics (MD) simulation

**Supporting Tables** ..........................................................................(S7–S8)
Table S1. ELP analogs synthesized in this study.
Table S2. Phase transition temperature values of ELP-azobenzene conjugates in phosphate buffer solutions.

**Supporting Figures** ..........................................................................(S9–S20)
Figure S1. UPLC-MS analysis of the synthesized ELP-azobenzene conjugates.
Figure S2. Correlation between the absorbance corresponding to the π-π* of each peptide and ratio of the *cis*-isomer.
Figure S3. Thermal stability of Azb-2(K-F2) under dark conditions.
Figure S4. Photo-isomerization of Azb-2(K-F2) upon UV-A light irradiation.
Figure S5. Correlation between ratio of the cis-isomer and the phase transition temperature ($T_t$).
Figure S6. Turbidity measurements of the ELP-azobenzene conjugates in phosphate buffer solutions.
Figure S7. Structure and turbidity measurements of Azb-2(F2).
Figure S8. Dynamic light scattering (DLS) autocorrelation curves.
Figure S9. Particle size distribution of Azb-2(F2).
Figure S10. Optical microscopy images of 2(F2-D)-Azb in PBS solution.
Figure S11. Scanning electron microscopy images of Azb-2(K-F2) prepared with irradiation of UV-A light.
Figure S12. Microscopy images of Azb-2(K-F2) aggregates containing Rhodamine B under dark conditions.

**Synthesis of elastin-like peptide analogs.**

Peptide synthesis was performed using the same method as previously reported.[1] Briefly, the short ELP analogs were synthesized by a CSBioII peptide synthesizer (Menlo Park, CA, USA). HBTU (0.45 M) and OxymaPure (0.45 M) in *N*,*N*-dimethylformamide (DMF) were used as the condensing agents for peptide synthesis in 0.25 mmol scale with a manufacture's standard synthesis program. After peptide chain elongation, the peptides were cleaved from the resin with a reagents cocktail containing 95% TFA, 2.5% TIS, and 2.5% $H_2O$. After cleavage of the peptide synthesized from the resin, the resulting mixture was poured into 50 mL of diethyl ether and centrifuged to separate peptide precipitates from the cocktail. The resulting peptide precipitate was resuspended in 50 mL of diethyl ether and the resulting solution was centrifuged to remove contaminants. The molecular weights of the peptides were confirmed by ACQUITY UPLC H-Class (Waters Co.) equipped with an ACQUITY UPLC BEH C-18 column (100 mm, flow rate 0.6 mL/min) (Waters Co.) at 49°C . The eluting product was detected by UV absorption at 225 nm and a quadrupole mass spectrometer, ACQUITY QDa (Waters Co.). The solvent system for UPLC consisted of 0.1% formic acid aqueous solution (v/v, solvent A) and 0.1% formic acid in acetonitrile (v/v, solvent B), and elution was performed with a linear gradient (24% to 56%) of solvent B over 4.23 min.

**Synthesis of ELP-azobenzene conjugates.**

*F2-Azb-F2NH$_2$ and F2-Azb-F2OH*: H-(FPGVG)$_2$-NH$_2$ and H-(FPGVG)$_2$-OH were synthesized on a 0.1 mmol scale using a CSBIO II peptide synthesizer. Fmoc-NH-SAL-MBHA resin and Fmoc-Gly-Wang resin were employed as the solid-phase supports for F2-Azb-F2NH$_2$ and F2-Azb-F2OH, respectively. Fmoc-protected 4-((4-aminopheyl)diazenyl)benzoic acid (0.3 mmol, 139 mg), HATU (0.6 mmol, 228 mg), HOAt (0.6 mmol, 85.3 mg), DIPEA (1.2 mmol, 209 µL), and DMF (2 mL) were added to the resin and stirred for 5 h to introduce azobenzene moiety to the *N*-terminus of (FPGVG)$_2$ analogs on resin (without cleaving the peptide from polymer supports). Then, Fmoc-protecting group on an amino group of azobenzene was removed by treatment with 20% piperidine (in DMF, 2.5 mL) for 10 minutes two times. Continuously, Fmoc-Gly was conjugated with azobenzene-(FPGVG)$_2$ on resin by using HATU and HOAt in the same manner. The remaining residues were elongated in the same manner as conventional peptide synthesis using the peptide synthesizer. Then, final cleavage and purification of the peptides were carried out as described above.

*Azb-2(F2)*: In a well-dried Schlenk flask, azobenzene-4,4'-dicarbonyl dichloride (25 µmol, 7.68 mg) was dissolved in 2 mL of dichloromethane under nitrogen atmosphere and

cooled to 0 °C with ice bath. Subsequently, solution of H-(FPGVG)$_2$-OH (50 μM in 3 mL of dichloromethane) and DIPEA (60 μM, 10.5 mL) was added dropwise to the flask. The resulting mixture was allowed to elevate to room temperature (25 °C) and stirred overnight. The progress of the reaction was monitored by UPLC-MS. After completion, the reaction mixture was concentrated *in vacuo*. The residue was purified by RP-HPLC.

*Azb-2(K-F2)*: In a well-dried Schlenk flask, azobenzene-4,4'-dicarbonyl dichloride (25 μmol, 7.68 mg) was dissolved in 2 mL of dichloromethane under nitrogen atmosphere and cooled to 0 °C with ice bath. Subsequently, solution of H-Lys(Fmoc)-(FPGVG)$_2$-OH (50 μM in 3 mL of dichloromethane) and DIPEA (60 μM, 10.5 mL) was added dropwise to the flask. The resulting mixture was allowed to elevate to room temperature (25 °C) and stirred overnight. The progress of the reaction was monitored by UPLC-MS. After completion, the reaction mixture was concentrated *in vacuo*. The residue was dissolved in pure water (50 mL) and washed with ethyl acetate (50 mL, three times). The obtained aqueous layer was concentrated *in vacuo*. The residue was purified by RP-HPLC.

*2(F2-D)-Azb*: 4,4'-Azodianiline was synthesized by a previously reported method.[2] 4,4'-Azodianiline (1.0 mmol, 0.212 g) was dissolved in 3.0 mL of DMF, followed by the addition of Fmoc-Asp(OtBu)-OH (2.2 mmol, 0.905 g), HBTU (3.0 mmol, 1.138 g), OxymaPure (3.0 mmol, 0.426 g), and DIPEA (3.0 mmol, 522 μL). The reaction mixture was stirred at 50 °C overnight to conjugate Asp with amino groups of azobenzene. Subsequently, piperidine (1.0 mL) was added to the reaction mixture to remove Fmoc protecting group. The reaction mixture was concentrated *in vacuo*. Then, residue was recrystallized from mixture solvent of dichloromethane and ethyl acetate to afford Azb-2(Asp(OtBu)) as yellow powder (250 mg, 0.451 mmol, 45.1% yield). Then, Azb-2(Asp(OtBu)) (25 μmol, 13.9 mg), Boc-protected (FPGVG)$_2$-OH (50 μmol, 51.6 mg), HBTU (0.1 mmol, 37.9 mg), OxymaPure (0.1 mmol, 14.2 mg), and DIPEA (0.2 mmol, 34.8 μL) were dissolved in 3.0 mL of dichloromethane and stirred at 25 °C for 5 h. The resulting mixture was added 2 mL of a mixture of 95% TFA/2.5% TIS/2.5% $H_2O$ and stirred further 1 h. The reaction mixture was concentrated *in vacuo* and purified by a Sep-Pak Vac 35 cc C18 cartridge and RP-HPLC.

**Purification of the peptides.**

Before final purification by reversed-phase (RP)-HPLC, the synthesized peptide analogs were pre-purified by using a Sep-Pak Vac 35 cc C18 cartridge (Waters Co., Milford, MA).[1] All peptides synthesized were dissolved in 15% acetonitrile aqueous solution and

applied to the Sep-Pak cartridge, and an acetonitrile aqueous solution was subsequently poured into the Sep-Pak cartridge as eluent. The eluting solution was fractionated every 50 mL. The concentration of acetonitrile in the eluent was gradually increased to 15%, 30%, 60%, and 99% to separate the peptides. The elution fractions containing the peptides were identified ACQUITY UPLC H-Class (Waters Co.). These fractions were evaporated and followed by lyophilization to obtain the peptide powder. Further purification was performed by RP-HPLC (JASCO PU-2089 equipped with UV-2075 or JASCO PU-4180 equipped with UV-4075, JASCO, Tokyo, Japan) using a C8 column (COSMOSIL 5C8-AR-300 Packed Column, 20 mmI.D. x 150 mm, 5 µm, 300 Å, Nacalai Tesque Inc., Kyoto, Japan). A solvent system consisting of 0.1% TFA aqueous solution (v/v, solvent A) and mixture of 80% acetonitrile and 20% solvent A (v/v, solvent B) were used for the gradient elution (with a linear gradient of solvent B 20% to 60% over 40 min and flow rate 3.5 ml/min). Purity and molecular weights of the peptides were confirmed by ACQUITY UPLC H-Class (Waters Co.) as shown above.

**Molecular Dynamics (MD) simulation**

MD simulation was carried out by using GROMACS 2019 software with Amber ff99SB-ILDN force field.[3] In this study, the structural dynamics of *trans*- and *cis*-isomers of F2-Azb-F2 were analyzed. TIP3P explicit solvent model was used to analyze the interactions between the peptides and water molecules. Initial conformations (.pdb file) were generated by Discovery studio 4.5 software (Dassault Systemes BIOVIA, San Diego, CA, USA) and translated into .gro file by gmx pdb2gmx command in GROMACS. The model of F2-Azb-F2 were placed in 7 × 7 × 7 $nm^3$ cubic box and solvated with explicit TIP3P water molecules by gmx solvate.

The simulation was performed at 278 and 303 K to consider the change in molecular structure due to difference of temperature. These systems contained two minimizations, heating step, equilibrium step, and production step. The first minimization was performed by steepest descent algorithm, using a maximum of 10,000 steps, the maximum step size (emstep) of 0.01 nm, the tolerance (emtol) of 10.0 kJ $mol^{-1}$ $nm^{-1}$, and no constraints; The second minimization was performed by conjugate gradient algorithm, using a maximum of 20,000 steps, emstep of 0.01 nm, emtol of 10.0 kJ $mol^{-1}$ $nm^{-1}$, and no constraints; heating step: parameters are 500,000 steps, time step 2 fs, initial temperature 0 K, target temperature (278/303 K), LINCS constraint to h-bond atoms, annealing type single, annealing npoints 2, and annealing time 0 200; an equilibration step: parameters are 500,000 steps with a time step of 2 fs, target temperature (278/303 K), LINCS constraint to h-bond atoms, and reference pressure 1.0 bar; and finally, a production step consisting

of 15,000,000 steps for both *trans*- and *cis*-F2-Azb-F2, time step 2 fs, target temperature (278/303 K), pressure coupling decay time (tau-p) = 1.0, LINCS constraint to h-bond atoms, nonbond list radius (rcoulomb, rvdw) = 1.0 nm, nonbond lower cutoff distance (rlist) = 1.0 nm, electrostatics = particle-mesh Ewald (PME) method, dynamics integrator= leapfrog, and random number seed = 1732. Trajectories (30,000 frames for each isomer) were processed by gmx trjcomv command to correct the break in the molecular structure due to periodic boundary condition. During this process, the number of frames were reduced to 1/10 (3,000 frames). Then, peptide structures were analyzed using the processed trajectory file with omitting first 10 ns.


1. Suyama, K.; Mawatari, M.; Tatsubo, D.; Maeda, I.; Nose, T. Simple regulation of the self-assembling ability by multimerization of elastin-derived peptide $(FPGVG)_n$ using nitrilotriacetic acid as a building block. *ACS Omega* **2021**, *6*, 5705–5716.
2. Naeimi, H.; Safari, J.; Heidarnezhad, A. Synthesis of Schiff base ligands derived from condensation of salicylaldehyde derivatives and synthetic diamine. *Dyes and Pigments* **2007**, *73*, 251–253.
3. Hess, B.; Kutzner, C.; van der Spoel, D.; Lindahl, E. GROMACS 4: Algorithms for Highly Efficient, Load-Balanced, and Scalable Molecular Simulation. *J. Chem. Theory Comput.* **2008**, *4* (3), 435–447. https://doi.org/10.1021/ct700301q.

**Table S1. ELP analogs synthesized in this study.**

| Peptide | Retention time (min) | | MS (QDa) *m/z* | | |
|---|---|---|---|---|---|
| | *cis*-isomer | *trans*-isomer | Composition formula | calculated | found |
| F2-Azb-F2 | 2.643 | 2.885 | $C_{105}H_{135}N_{23}O_{22}$ | 1036.51 $[M+2H]^{2+}$ | 1036.72 |
| F2-Azb-F2-$NH_2$ | 2.532 | 2.739 | $C_{105}H_{135}N_{24}O_{21}$ | 1036.02 $[M+2H]^{2+}$ | 1036.38 |
| Azb-2(F2) | 3.687 | 4.047 | $C_{106}H_{134}N_{22}O_{24}$ | 1051.00 $[M+2H]^{2+}$ | 1051.09 |
| Azb-2(K-F2) | 1.793 | 1.965 | $C_{118}H_{158}N_{26}O_{26}$ | 1179.10 $[M+2H]^{2+}$ | 1179.29 |
| 2(F2-D)-Azb | 1.727* | | $C_{112}H_{146}N_{26}O_{26}$ | 1137.05 $[M+2H]^{2+}$ | 1137.09 |

Retention times of each peptide were determined by RP-UPLC-MS.

*: The *trans*- and *cis*-isomer of 2(F2-D)-Azb could not be divided by UPLC.

**Table S2. Phase transition temperature values of ELP-azobenzene conjugates in phosphate buffer (PB) solutions.**

| Peptide | Concentration | Solvent | $T_{t,\ dark}$ (°C) | $T_{t,\ UV}$ (°C) |
|---|---|---|---|---|
| Azb-2(K-F2) | 3.00 mg/mL (1.27 mM) | PB (pH 7.4) | 27.57 ± 0.18 | 31.42 ± 0.78 |
| Azb-2(K-F2) | 3.00 mg/mL (1.27 mM) | PB (pH 2.1) | Not determined | - |
| Azb-2(K-F2) | 3.00 mg/mL (1.27 mM) | PB (pH 11.5) | Not determined | - |
| 2(D-F2)-Azb | 1.50 mg/mL (0.66 mM) | PB (pH 7.4) | 13.13 ± 0.53 | Not determined |
| 2(D-F2)-Azb | 1.50 mg/mL (0.66 mM) | PB (pH 2.1) | Not determined | - |
| 2(D-F2)-Azb | 1.50 mg/mL (0.66 mM) | PB (pH 11.5) | Not determined | - |

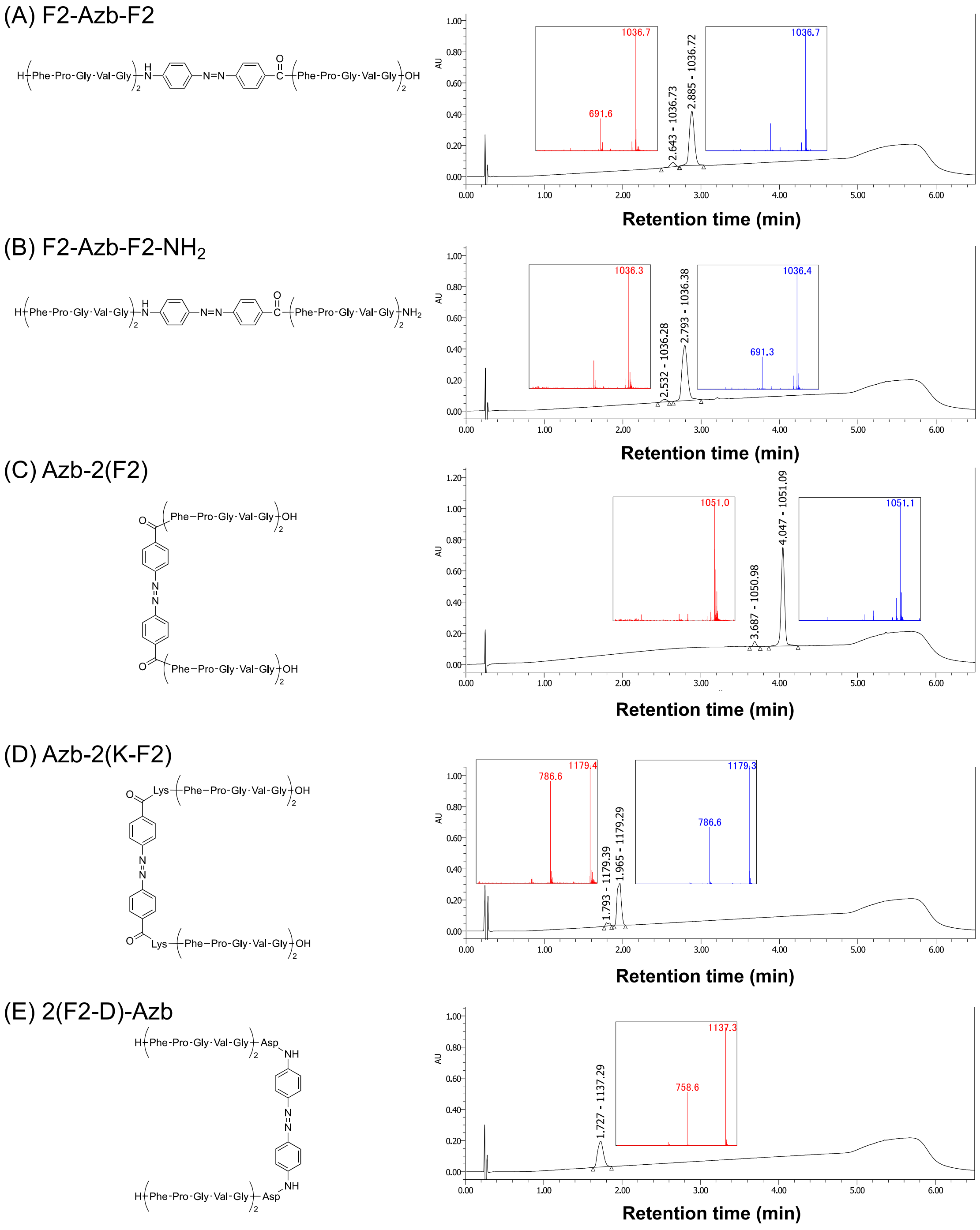


**Figure S1. UPLC-MS analysis of the synthesized ELP-azobenzene conjugates.** UPLC data of the synthesized peptide are shown. (A) F2-Azb-F2, (B) F2-Azb-F2-$NH_2$, (C) Azb-2(F2), (D) Azb-2(K-F2), and (E) 2(F2-D)-Azb. Inset in each panel shows MS spectra of the *cis*-isomer (red) and the *trans*-isomer (blue) determined by ACQUITY QDa mass spectrometer. The *cis*- and *trans*-isomers of 2(F2-D)-Azb could not be divided.

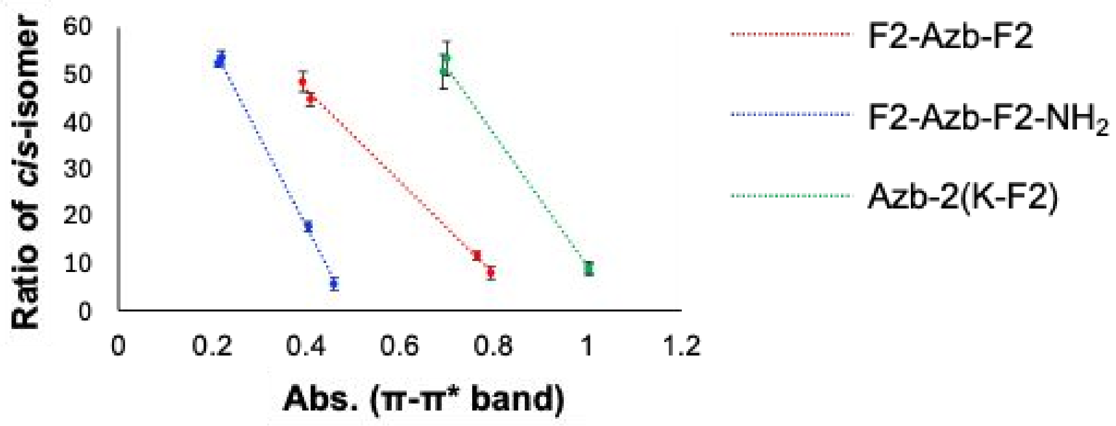


**Figure S2. Correlation between the absorbance corresponding to the π-π* of each peptide and ratio of the *cis*-isomer.**

Each peptide solution was prepared in the PBS solution at a concentration of 50 μM.

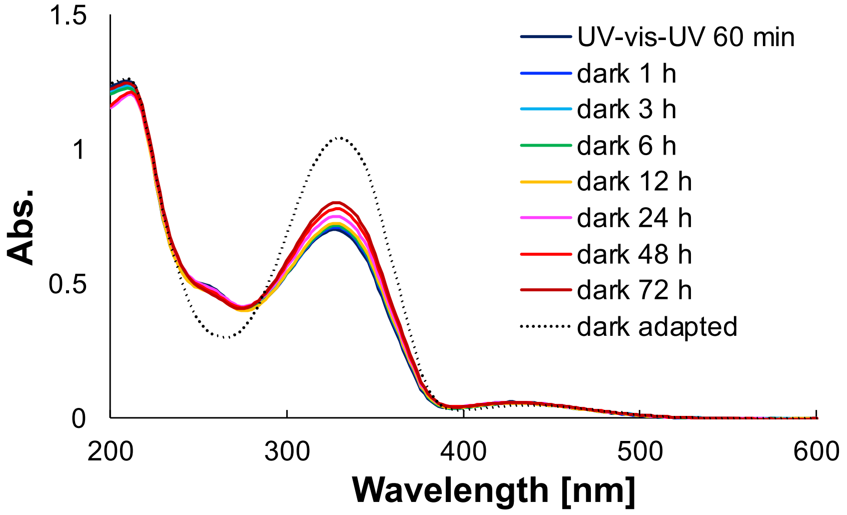


**Figure S3. Thermal stability of Azb-2(K-F2) under dark conditions.**
Time-dependent UV-vis spectral changes of Azb-2(K-F2) in PBS solution (50 μM) at 37 °C under dark conditions after irradiation with UV light. Dotted line: UV-vis spectra of dark-adapted solutions (i.e., before UV light irradiation) of Azb-2(K-F2).

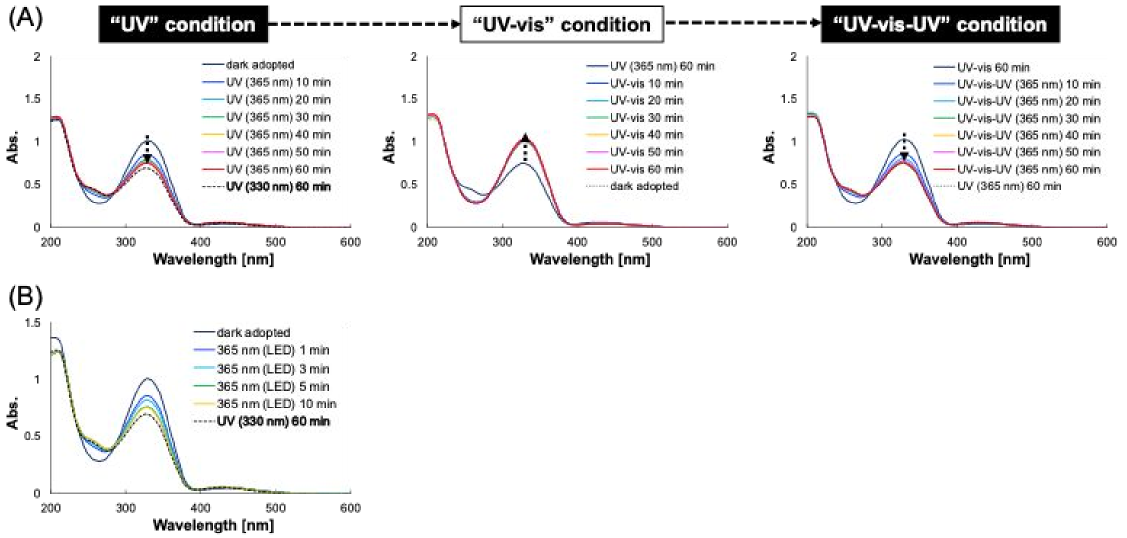


**Figure S4. Photo-isomerization of Azb-2(K-F2) upon UV-A light irradiation.**

(A) Time-dependent UV-vis spectral changes of Azb-2(K-F2) upon sequential irradiation with UV-A light (365 nm), visible light, and UV-A light (365 nm). The peptide solution was prepared in the PBS solution at a concentration of 50 μM and irradiated with UV-A light with wavelength of 365 nm by using The Xe-W lamp of a JASCO FP8500 spectrophotometer (JASCO Co.) at 5 °C. (B) Time-dependent UV-vis spectral changes of Azb-2(K-F2) in the PBS solution at a concentration of 50 μM. The peptide solution was irradiated with UV-A light with wavelength of 365 nm by using Mounted LED M365L3 at 5 °C. The trace of the "UV 60 min" sample irradiated with UV light (330 nm) is shown as a dashed black line in the "UV" panels in (A) and panel (B) as a reference.

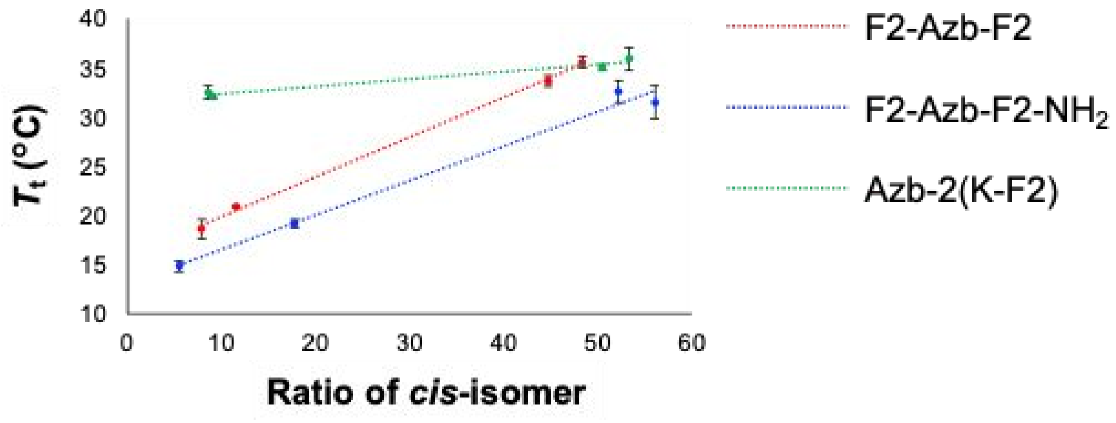


**Figure S5. Correlation between ratio of the *cis*-isomer and the phase transition temperature ($T_t$).**

F2-Azb-F2 (red line, 100 μM), F2-Azb-F2-NH2 (blue line, 50 μM), and Azb-2(K-F2) (green line, 1.27 mM).

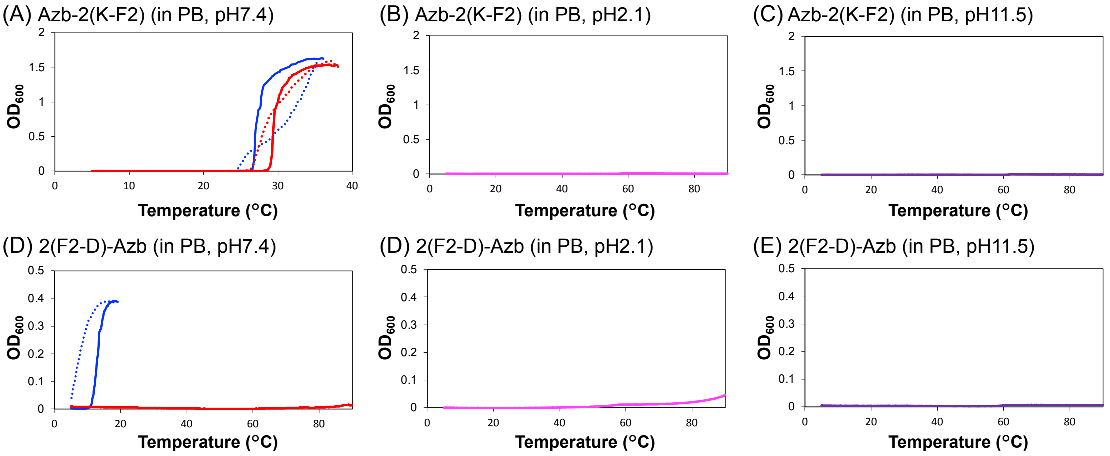


**Figure S6. Turbidity measurements of the ELP-azobenzene conjugates in phosphate buffer solutions.**

Turbidity changes of the ELP-azobenzene conjugates in phosphate buffer solutions associated with heating (solid lines) and cooling (dashed lines). (A) Azb-2(K-F2) (1.27 mM in phosphate buffer, pH 7.4), (B) Azb-2(K-F2) (1.27 mM in phosphate buffer, pH 2.1), (C) Azb-2(K-F2) (1.27 mM in phosphate buffer, pH 11.5), (D) 2(F2-D)-Azb (0.66 mM in phosphate buffer, pH 7.4 and pH 2.1), and (E) 2(F2-D)-Azb (0.66 mM in phosphate buffer, pH 11.5). Turbidity measurements were carried out for dark-adapted solution (blue lines) and after UV irradiation (red lines) in (A) and (D).

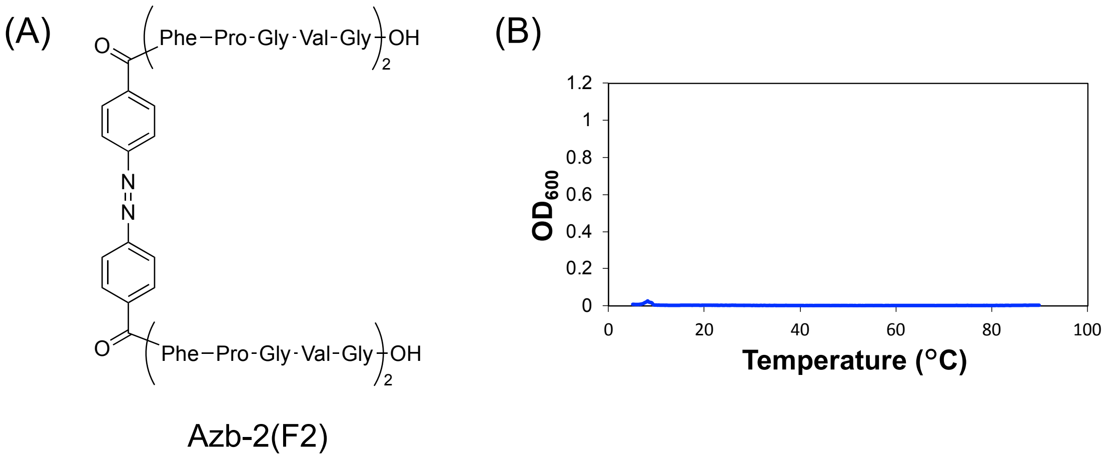


**Figure S7. Structure and turbidity measurements of Azb-2(F2).**

(A) Chemical structure of Azb-2(F2), an analog of Azb-2(KF2) in which the lysine residues were removed. (B) Turbidity changes of Azb-2(F2) in PBS solution (2.38 mM, 5.00 mg/mL) associated with heating. Turbidity measurements were carried out for dark-adapted solution.

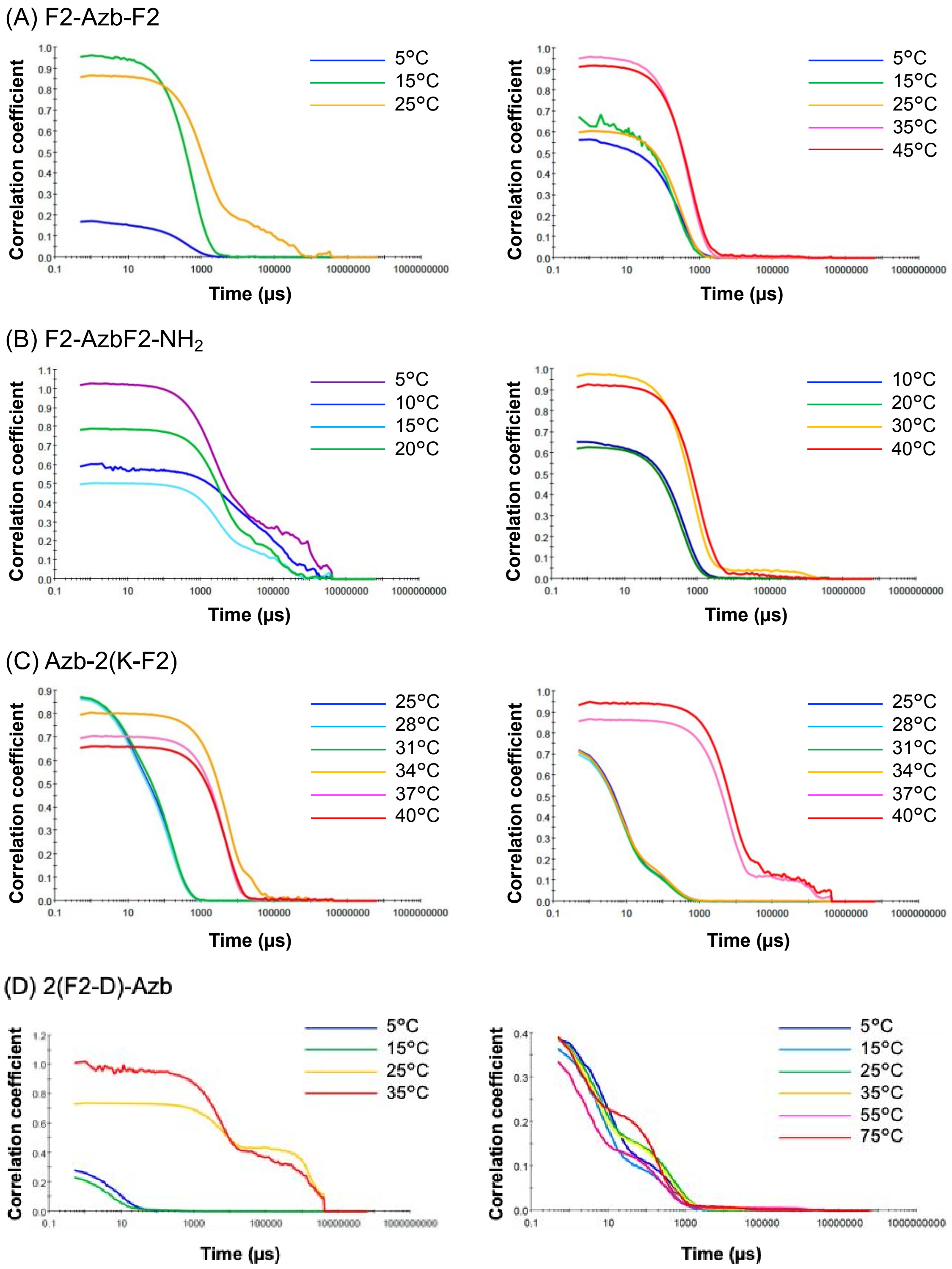


**Figure S8. Dynamic light scattering (DLS) autocorrelation curves.**

(A) F2-Azb-F2, (B) F2-Azb-F2-$NH_2$, (C) Azb-2(K-F2), and (D) 2(F2-D)-Azb before (left) and after (right) irradiation of UV light corresponding to each π-π* transition band for 1 h.

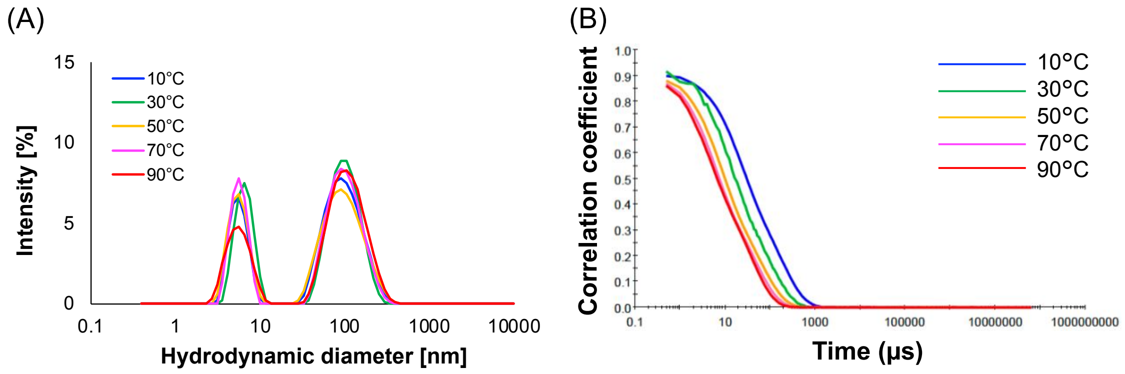


**Figure S9. Particle size distribution of Azb-2(F2).**

(A) Particle size distributions of Azb-2(F2) in PBS solution at a concentration of 5 mg/mL.

(B) Corresponding DLS autocorrelation curves.

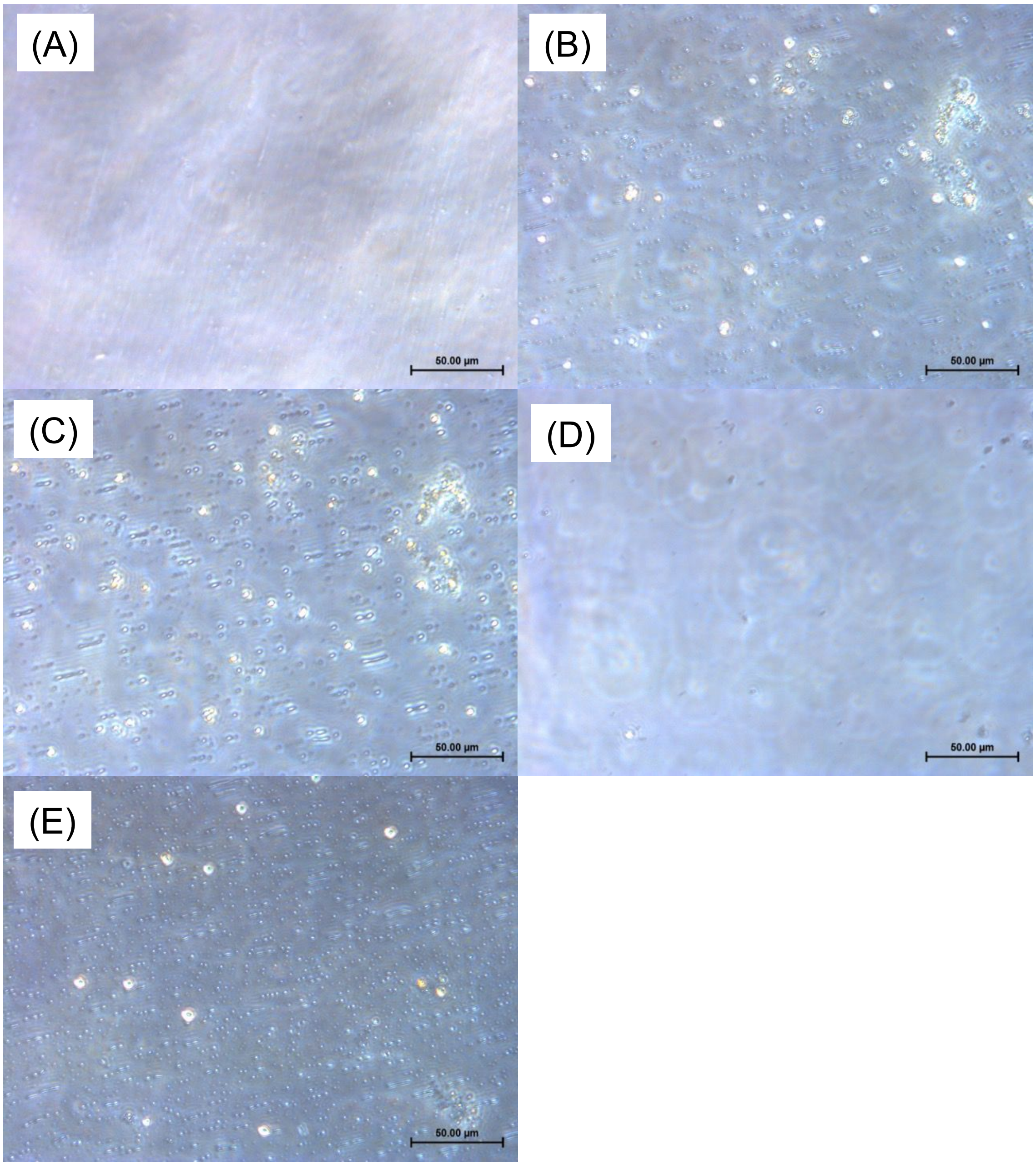


**Figure S10. Optical microscopy images of 2(F2-D)-Azb in PBS solution.**

Optical microscopic studies of the PBS solution of 2(F2-D)-Azb (1.5 mg/mL) at (A) 5 °C, (B) 25 °C, and (C) after incubation under 25 °C for 1 h. (D) After UV-A light irradiation (365 nm) for 5 min at 25 °C. (E) After incubation under room light for 15 min at 25 °C subsequent to (D). The magnification of all the images is 40×. Scale bars indicate 50 µm.

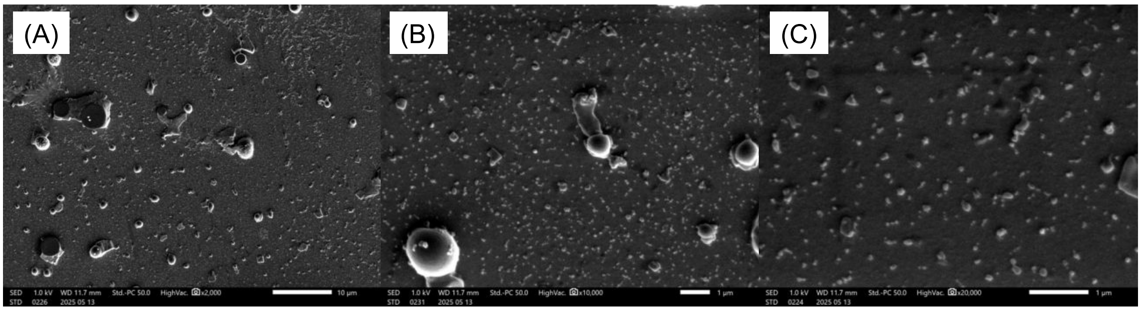


**Figure S11. Scanning electron microscopy images of Azb-2(K-F2) prepared with irradiation of UV-A light.**

Scanning electron microscopy images of Azb-2(K-F2) incubated at 37 °C with irradiation of UV-A light (365 nm). The magnification of the images is 2,000× for (A), 10,000x for (B), and 20,000 for (C). Scale bars represent 10 μm in (A), and 1 μm in (B) and (C).

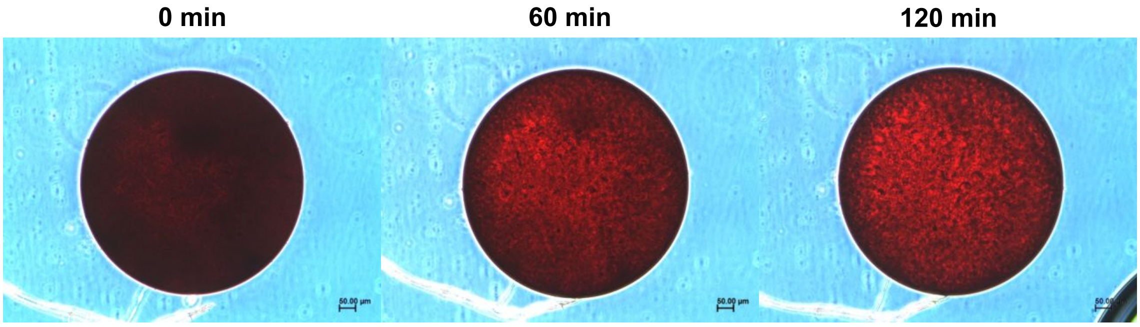


**Figure S12. Microscopy images of Azb-2(K-F2) aggregates containing Rhodamine B under dark conditions.**

Optical microscopic images of Azb-2(K-F2) aggregate containing Rhodamine B in PBS solution under dark condition. Time-cause observations of a prepared sample were carried out at 37 °C. The magnification of all the images is 10×. Scale bars represent 50 μm.
